# Characterization of microtubule-associated protein tau isoforms and Alzheimer’s disease-like pathology in normal sheep (*Ovis aries*): relevance to their potential as a model of Alzheimer’s disease

**DOI:** 10.1007/s00018-022-04572-z

**Published:** 2022-10-21

**Authors:** Emma S. Davies, Russell M. Morphew, David Cutress, A. Jennifer Morton, Sebastian McBride

**Affiliations:** 1 Department of Life Sciences, Aberystwyth University, Aberystwyth, UK; 2 Department of Physiology, Development and Neuroscience, University of Cambridge, Cambridge, UK

**Keywords:** Alzheimer’s disease, Amyloid-β, MAPT, Tau, Isoform expression, Sheep

## Abstract

Alzheimer’s disease is a chronic neurodegenerative disease that accounts for up to 80% of all dementias. Characterised by deteriorations of memory and cognitive function, the key neuropathological features are accumulations of β-amyloid and hyperphosphorylated tau, as ‘plaques’ and ‘tangles’, respectively. Despite extensive study, however, the exact mechanism underlying aggregate formation in Alzheimer’s disease remains elusive, as does the contribution of these aggregates to disease progression. Importantly, a recent evaluation of current Alzheimer’s disease animal models suggested that rodent models are not able to fully recapitulate the pathological intricacies of the disease as it occurs in humans. Therefore, increasing attention is being paid to species that might make good alternatives to rodents for studying the molecular pathology of Alzheimer’s disease. The sheep (*Ovis aries*) is one such species, although to date, there have been few molecular studies relating to Alzheimer’s disease in sheep. Here, we investigated the Alzheimer’s disease relevant histopathological characteristics of 22 sheep, using anti-β-amyloid (Abcam 12267 and mOC64) and phosphorylation specific anti-tau (AT8 and S396) antibodies. We identified numerous intraneuronal aggregates of both β-amyloid and tau that are consistent with early Alzheimer’s disease-like pathology. We confirmed the expression of two 3-repeat (1N3R, 2N3R) and two 4-repeat (1N4R, 2N4R) tau isoforms in the ovine brain, which result from the alternative splicing of two tau exons. Finally, we investigated the phosphorylation status of the serine396 residue in 30 sheep, and report that the phosphorylation of this residue begins in sheep aged as young as 2 years. Together, these data show that sheep exhibit naturally occurring β-amyloid and tau pathologies, that reflect those that occur in the early stages of Alzheimer’s disease. This is an important step towards the validation of the sheep as a feasible large animal species in which to model Alzheimer’s disease.

**Supplementary Information:**

The online version contains supplementary material available at 10.1007/s00018-022-04572-z.

## Introduction

Alzheimer’s disease (AD) is a progressive neurodegenerative disease that presents with debilitating memory and cognitive impairments, and accounts for between 60 and 80% of all dementias worldwide [100]. Whilst the prevalence and the consequent economic and social impacts of AD are predicted to increase each year as the global population ages, the exact aetiology of AD is still unknown, making the development of effective treatments extremely challenging [20]. Accumulations of β-amyloid (Aβ) exhibited as extracellular plaques and cerebral amyloid angiopathy (CAA), and accumulations of hyperphosphorylated tau present as paired helical filaments (PHFs) and neurofibrillary tangles (NFTs) are accompanied by significant neuronal loss and constitute the primary histopathological hallmarks of the disease [11, 20].

One particular challenge of using rodent species for preclinical studies is that rodents do not naturally exhibit either Aβ or tau pathology as they age [26]. To overcome this obstacle, it has been necessary to create transgenic mouse models overexpressing Aβ [68]. Such models have been used to elucidate the significance of mutations in the amyloid precursor (APP), and presenilin proteins (PSEN1 and PSEN2) that cause familial AD (FAD) [26]. Mutations in these proteins affect APP metabolism, leading to an increased production of the amyloidogenic form of Aβ (Aβ1–42), and impaired Aβ clearance [38]. These events form the basis of the ‘amyloid cascade hypothesis’ to explain the pathogenesis of AD, in which it is proposed that accumulation of Aβ1–42 is the primary pathological event that drives all other associated pathologies (including tau pathology, inflammation, vascular damage, and neuronal loss) [36]. This hypothesis, however, does not explain the mechanisms by which soluble and/or insoluble forms of intracellular and/or extracellular aggregates of Aβ and tau differentially affect one another [50, 75]. Indeed, while transgenic mouse models using APP and PSEN mutations present with significant Aβ pathology between three and six months of age they often fail to exhibit significant tau pathology or neuron loss [26]. Consequently, tau pathologies within these rodent models are achieved only by introducing tau mutations that cause tau pathology in other dementias, namely those associated with frontotemporal dementia with parkinsonism-17 (FTDP-17) [26]. This, combined with the relatively short lifespan of rodents means that the capacity of transgenic mouse models to fully reflect the aetiological mechanisms and temporal progression of familial AD is limited [81]. Furthermore, given the lack of naturally occurring age-related neuropathology, the ability of mouse models to recapitulate mechanisms associated with ‘sporadic’ AD (that is not associated with mutations in any of the FAD genes and accounts for more than 95% of AD cases), is also extremely restricted [22].

Increasing recognition of the limitations associated with rodent models of AD has led to the investigation of species that have a longer lifespan, a more physically and functionally differentiated brain, and a propensity to naturally develop both Aβ and tau pathology with age [23, 80]. Non-human primates such as the chimpanzee (*Pan troglodytes*), rhesus macaque (*Macaca mulatta*) and common marmoset (*Callithrix jacchus*) are of particular interest, because they naturally develop some AD-like pathology [41]. For example, diffuse and dense-cored Aβ plaques, and CAA have been detected in aged chimpanzees [25, 28, 30, 78], rhesus macaques [79, 96] and marmosets [35, 58], with quantities of Aβ in aged individuals comparable to levels seen in AD patients. NFTs have also been described within the entorhinal cortex of aged chimpanzees and rhesus macaques [4], while in marmosets, abnormally phosphorylated tau has been identified as early as adolescence [77]. A range of other species have been found to exhibit spontaneous AD-like pathology. Such species include the gorilla (*Gorilla gorilla gorilla* [71] and *Gorilla beringei beringei* [72])*,* Ursidae species [16, 88, 95], cetacean species [88], and pinniped species [92]. The domestic dog (*Canis familiaris*) [17, 18, 80, 86] and cat (*Felis catus*) [12] also develop cognitive decline alongside diffuse Aβ plaques in old age. However, tau pathology rarely accompanies Aβ deposition and dense-cored Aβ plaques and NFTs are not consistently detected. Whilst non-human primates, dogs and cats have the potential to mitigate the limitations of current rodent AD models, they are limited in terms of their ethical use and, therefore, their numbers available to facilitate robust experimental design.

We chose to focus on sheep as a potential animal model of human AD for a number of reasons. Sheep have a moderate lifespan (15–20 years), an extensively annotated reference genome [49], are numerous within a well-established agricultural production infrastructure, and can be kept in a naturalised environment. Sheep also have a highly gyrencephalic neocortex with differentiated cortical and subcortical structures [61]. This species has also been used as a model for studying the neurodevelopmental and cognitive effects of hormone manipulation [45, 76], Huntington’s disease [46, 62, 63, 85], and Batten disease [6, 24, 82]. Sheep between 8 and 14 years of age have also been shown to spontaneously develop both diffuse Aβ plaques [74] and NFTs [7, 64, 65]. These findings were confirmed in a pilot study (within this study) and suggest that sheep could provide a more robust model of AD than species that do not naturally develop this pathology. Few molecular studies of AD-relevant proteins have been conducted in sheep to fully assess the translatability of the model. For example, whereas humans express six central nervous system (CNS) tau isoforms formed from the alternative splicing of three exons, the tau isoform expression of sheep has been predicted but not confirmed experimentally [47, 66]. Furthermore, whilst in humans over 70 different possible phosphorylation sites of the tau protein have been identified and associated with different time points of AD progression [93], similar information (that may be critical in identifying early tau dysfunction during AD aetiology) is extremely limited in the ovine model of AD pathology [7, 64, 65]. The aims of this study were to characterise Aβ and tau pathology, including tau phosphorylation, in sheep of various ages, and to investigate tau isoform expression in sheep. The results of the pilot study are also described.

## Methods

### Pilot experiment

The methodology of the pilot study which examined the brains of two sheep, one 21 years of age (P1), and one 16 years of age (P2) is described within the supplementary material (Online Resource 1). These sheep had been euthanised by a veterinary surgeon for humane reasons and their brains were donated by the owner.

### Main sheep

A total of 30 sheep brains, obtained as by-products from commercial sheep intended for human consumption (hereafter called stock sheep) were examined in this study. All these sheep were killed by electrical stunning and exsanguination. The breed of each animal was recorded and its age (determined via dentition [15]) was estimated at the point of sample collection. Sheep were judged to be purebred (Cheviot *n* = 1, Southdown *n* = 1, Herdwick *n* = 1), Mule (*n* = 15), or Texel cross (*n* = 12). All but one of the sheep were female. Sheep were estimated to be 1–2 years of age (*n* = 10), 2–3 years of age (*n* = 10), and > 5 years of age (*n* = 10).

### Histopathology

Twenty brains (from stock sheep aged 1–2 and > 5 years of age) were examined histologically. Following extraction, the right brain hemisphere was dissected, snap frozen, and stored at − 80 °C until use. The left brain hemisphere was fixed in 10% neutral-buffered formalin (4% v/v formaldehyde) for 4 days, dissected into coronal blocks and post-fixed for a further 3 days. Fixed tissue was dehydrated and embedded in paraffin wax, cut into 4 μm thick sections, mounted onto slides, and dried at 45* °*C overnight, before being bonded at 70* °*C for 1 h*.* Sections were stained using haematoxylin and eosin (CellPath). Additional 4 μm thick human hippocampal tissue samples were obtained from the Medical Research Council UK Brain Bank Network [the South West Dementia Brain Bank (SWDBB)]. The tissue sections were histologically evaluated by the SWDBB and had a diagnosis of severe AD (AD positive) or were described as having no significant neuropathological abnormalities (AD negative). These tissue samples were used as controls for antibody staining (see Online Resource 1).

### Immunohistochemistry

Immunohistochemistry was performed using a labelled streptavidin–biotin method. Rinses between incubations were conducted using tris buffered saline (TBS). Briefly, following deparaffinisation and rehydration, antigen retrieval was performed by incubating sections in 95% v/v formic acid for 20 min (RT). The sections were incubated in 5% v/v normal goat serum (Merck, S26-100) with 1% w/v BSA protein block for 2 h (RT) to prevent non-specific binding. Streptavidin and biotin blocking (Vector laboratories SP-2002) was conducted to prevent endogenous biotin binding. Sections were incubated in a primary antibody (Table 1) overnight at 4* °C*. Antibodies included: rabbit polyclonal anti-Aβ1–42 (Abcam, ab12267, specific to the Aβ C-terminus, 1:300), rabbit recombinant anti-Aβ1–42 (mOC64, Abcam, ab201060, specific to aggregated Aβ at residues 3-EFRH-6 [39, 67], 1:800), rabbit recombinant anti-phospho-tau serine396 (S396, Abcam, ab109390, 1:6000), and mouse monoclonal anti-phospho-tau serine202/threonine205 (AT8, Thermo-Fisher, MN1020, 1:100). A rabbit polyclonal anti-tau (DAKO #A0024, 1:10,000), was used on select sections to confirm the labelling of tau observed with phospho-tau antibodies. Sections were incubated in 0.3% hydrogen peroxide for 10 min (RT) to prevent endogenous peroxide activity. Sections were incubated in the appropriate polyclonal biotinylated IgG goat anti-mouse or anti-rabbit secondary antibody (ThermoFisher Scientific, #62-6540, #65-6140, 1:300) with 1% w/v BSA for 1 h (RT). Sections were incubated in streptavidin HRP (Abcam, ab64269) for 10 min (RT) and 3,3′-diaminobenzidine (DAB, Abcam, ab64238) for 5 min (RT), before being counterstained in haematoxylin. Negative control slides, omitting the primary antibody, and control slides where tissue was incubated directly in streptavidin and biotin, and DAB were prepared to determine the presence of any non-specific interactions.

**Table 1:** Summary of antibodies used within immunohistochemistry procedures

Antigen	Antibody	Dilution	Company
Aβ1–42 (ab12267)	Rabbit polyclonal to the Aβ1–42 C-terminus	1:300	Abcam, ab12267
Aβ1–42 (mOC64)	Rabbit monoclonal recombinant to aggregated Aβ residues 3-EFRH-6	1:800	Abcam, ab201060
Phosphorylated-tau (AT8)	Mouse monoclonal to phospho-tau serine202 & threonine205	1:100	Thermo-Fisher, MN1020
Phosphorylated-tau (S396)	Rabbit monoclonal recombinant to phospho-tau serine396	1:6000	Abcam, ab109390
Pan-tau (DAKO)	Rabbit polyclonal to the tau C-terminus	1:10,000	DAKO #A0024

### Protein extraction

Protein samples from the frontal cortex, entorhinal cortex and hippocampus were extracted from each of the stock sheep brains (*n* = 30) used in this study. Tissue was homogenised in 10 volumes of homogenisation buffer (TBS (50 mM Tris/HCl, pH 7.4) with 1 tablet of cOmplete™, Mini EDTA-free Protease Inhibitor Cocktail (Sigma-Aldrich^®^), and 1 tablet of PhosSTOP EASYpack (Sigma-Aldrich^®^) phosphate inhibitor. Homogenised tissue was centrifuged at 27,000×*g* for 1 h. The supernatant was collected as the soluble fraction and stored at − 80 °C until use.

### Western blot

Soluble protein sample concentrations were quantified using the Bradford Reagent assay. An aliquot (10 μg) of each sample was re-suspended in loading buffer (0.2 M Tris–HCl (pH 6.8), 8% w/v sodium dodecyl sulfate, 40% w/v glycerol, 0.02% bromophenol blue) with 50 mM dithiothreeitol (DTT)) and boiled at 95 °C for 10 min. Samples were separated on a 10% acrylamide gel using a mini-Protean (Biorad) kit, equilibrated with TGS gel running buffer (Biorad: 25 mM Tris, 192 mM glycine, 0.1% w/v SDS, pH 8.3). Proteins were transferred to a nitrocellulose membrane (0.45 μm pore size, Amersham™, Protran^®^). Membranes were blocked with 5% w/v skimmed milk in Tween 20 tris buffered saline (TTBS) overnight at 4 °C. To detect phosphorylated tau, membranes were incubated in recombinant rabbit anti-phospho-tau serine396 antibody (S396, Abcam, 1:10,000). A human tau protein ladder (Merck, Sigma-Aldrich, T7951) comprised of six recombinant human tau isoforms was used as a non-phosphorylated tau control to ensure that the S396 antibody did not detect non-phosphorylated tau. To detect total-tau and the recombinant human tau ladder, membranes were incubated in mouse anti-human pan-tau-46 monoclonal antibody (Thermo-Fisher, 1:1000). The membranes were incubated in the appropriate IgG alkaline phosphatase conjugated secondary antibody (1:30,000) for 1 h (RT) and were developed for 2 min using the BCIP/NBT (5-Bromo-4-chloro-3-indolyl phosphate/nitro blue tetrazolium) system. Images were captured using a GS-800 calibrated densitometer (Biorad) and signal intensity was analysed with ImageQuant™ software. For each blot, the area of measurement was standardised, and the background signal was controlled for using the local median value.

### Plasmid cloning PCR and sequence analysis

For the molecular analysis and isoform expression characterisation of tau, total RNA was extracted from the frontal cortex of one stock sheep (sheep #1) using a Direct-zol™ RNA Microprep Kit (Zymo Research). Complimentary DNA (cDNA) was created using the High Capacity RNA-to-cDNA™ Kit (Applied biosystems, Thermofisher Scientific). cDNA was used for subsequent PCR using three pairs of primers designed using the NCBI Predicted *Ovis aries* microtubule associated protein tau (MAPT) transcript, variant X1, XM_027974371.1 (Online Resource 1; table S1). PCR conditions consisted of 95 °C for 3 min, followed by 45 cycles of 95 °C for 30 s, 61 °C for 1 min, and 72 °C for 2 min. A final extension of 30 min was used. PCR products were separated using a 2% agarose gel. Gels were visualised and analysed using a Typhoon FLA laser scanner (GE Healthcare). PCR products were excised from the gel and recovered using an ISOLATE II PCR and Gel Kit (Bioline). PCR products were ligated into a plasmid vector and transformed into Alpha-select bronze efficiency competent cells (Bioline) using the pGEM^®^-T Easy Vector system (Promega). Each transformation culture was plated onto ampicillin/IPTG/X-Gal LB-agar plates and incubated overnight at 37 °C. Transformed colonies were picked and resuspended in 50 μl of nuclease free water. 10 μl was used for colony PCR and analysed using gel electrophoresis. Colonies with an appropriately sized insertion were cultured in 7 ml of LB broth (containing 100 mg/ml ampicillin) overnight (37 °C, 200 rpm). Plasmids were purified from the cell culture using an Isolate II Plasmid Mini Kit (Bioline) and underwent Sanger sequencing at the Translational Genomics Facility, Aberystwyth University. Multiple sequence alignments were performed using BioEdit Sequence Alignment Editor software [36]. Exons are described using the non-standard nomenclature system used by Buée et al. [10], rather than the standard nomenclature system described by Sündermann et al*.* [90].

### Mass spectrometric analysis

Brain tissue samples (10 μg) from four stock sheep > 5 years of age (sheep #1, 2, 3, 4) were separated by SDS-page electrophoresis using a 10% acrylamide gel. Five protein bands from each sample, which corresponded to the phospho-tau bands identified on western blot membranes were excised from the acrylamide gel. Briefly, gel pieces were de-stained in 50% v/v 50 mM ammonium bicarbonate (pH 8) and 50% v/v acetonitrile (30 min at 37 °C) until clear. Samples were dehydrated with 100% v/v acetonitrile (15 min at 37 °C) and air dried (50 °C). Samples were incubated with 100 μl, 10 mM DTT in ammonium bicarbonate (30 min 80 °C). Samples were incubated with 55 mM IAA in ammonium bicarbonate (20 min, RT), and washed twice with 50% v/v ammonium bicarbonate and 50% v/v acetonitrile (15 min, RT). Samples were dehydrated, air dried, and rehydrated in 50 mM ammonium bicarbonate containing 10 ng/μl trypsin (modified trypsin sequencing grade, Promega), overnight at 37 °C. Samples were eluted in 20–50 μl of water, followed by 50% v/v acetonitrile, with 5% v/v formic acid. Elutant was dried and stored at − 20 °C. Samples were re-constituted using 20 μl 0.1% v/v formic acid immediately before analysis.

Liquid chromatography mass spectrometry was conducted using an Orbitrap Fusion™ Tribrid™ mass spectrometer (Thermo Scientific™), with H-ESI ion source, coupled to an UltiMate™ 3000 UHPLC tower (Dionex, Thermo Scientific ™) comprised of a rapid separation pump, column compartment and auto-sampler. Liquid chromatography was conducted with an Agilent eclipse plus C18 column (2.1 × 5 mm, with 1.8 μM particle size). The mobile phases for gradient elution were ultrapure water (18.2 $\Omega$) with 0.1% formic acid as elutant 1, and 95% aceronitrile with 0.1% formic acid as elutant 2. Liquid chromatography was performed with a flow rate of 0.1 ml/min starting with elutant 2 at 3–40% for 9 min, 40–100% for 2 min, and 100% for 1 min, before equilibration at 3% for 1.5 min. Ions were generated in a H-ESI source with a source voltage of 3500 V in positive mode, sheath gas: 25, aux gas: 5, a vaporiser temperature of 75 °C and an ion transfer temperature of 275 °C. Standard peptide analysis parameters were used; parent ions were detected in profile mode in the 375–1500 *m*/*z* range in the orbitrap at a resolution of 120,000 and a maximum injection time of 50 ms in positive mode. MS2 data were collected in data-dependent mode including charge states of 2–7. Dynamic exclusion of masses was conducted for 20 s after initial selection for MS2. Ions were formed by fragmentation by collision induced dissociation with a collision energy of 35%. Resulting ions were detected in the Ion Trap in centroid mode.

Mass spectral data were submitted to the MASCOT (http://www.matrixofscience.com) for database comparison. Spectre were searched against the Ensembl *Ovis Aries* (Oar_v3.1) protein database. Search parameters allowed up to one missed cleavage and included a fixed peptide modification of carbamidomethylation and a variable modification of oxidised methionine residues. Peptide charges 2+, 3+ and 4+ were selected for analysis. Parameters also included a set peptide tolerance of ± 1.2 Da and a MS/MS tolerance of ± 0.6 Da. Individual sequences were compared against a transcript protein map generated using the NCBI Predicted *Ovis aries* MAPT transcript, variant X1, XM_027974371.1, and the NCBI sequence, XP_027830172.1.

### Statistical analysis

Twenty of the brains (from stock animals 1–2 and > 5 years of age) were examined using immunohistochemistry. The number of positive regions labelled using the AT8 antibody were verified via microscopy and manually counted. Samples from all brains in the main study were used for western blot analysis. The relative intensities of the positive signals, probed with the anti-phospho-tau antibody, S396, were quantified using ImageQuant™ software. The average signal intensities were measured, standardised, and the background signal was controlled for using the local median value. As on balance, both of these data (average number of AT8-positive regions identified and western blot signal intensity) met the assumptions for parametric statistical analysis, the effect of age, brain region and the interaction of these two factors on the data were analysed using a two-way analysis of variance (ANOVA), with Tukey HSD post hoc testing. Statistical significance was determined if *p* ≤ 0.05. Data are reported as mean ± SEM.

## Results

### Pilot study

**Fig. 1:**
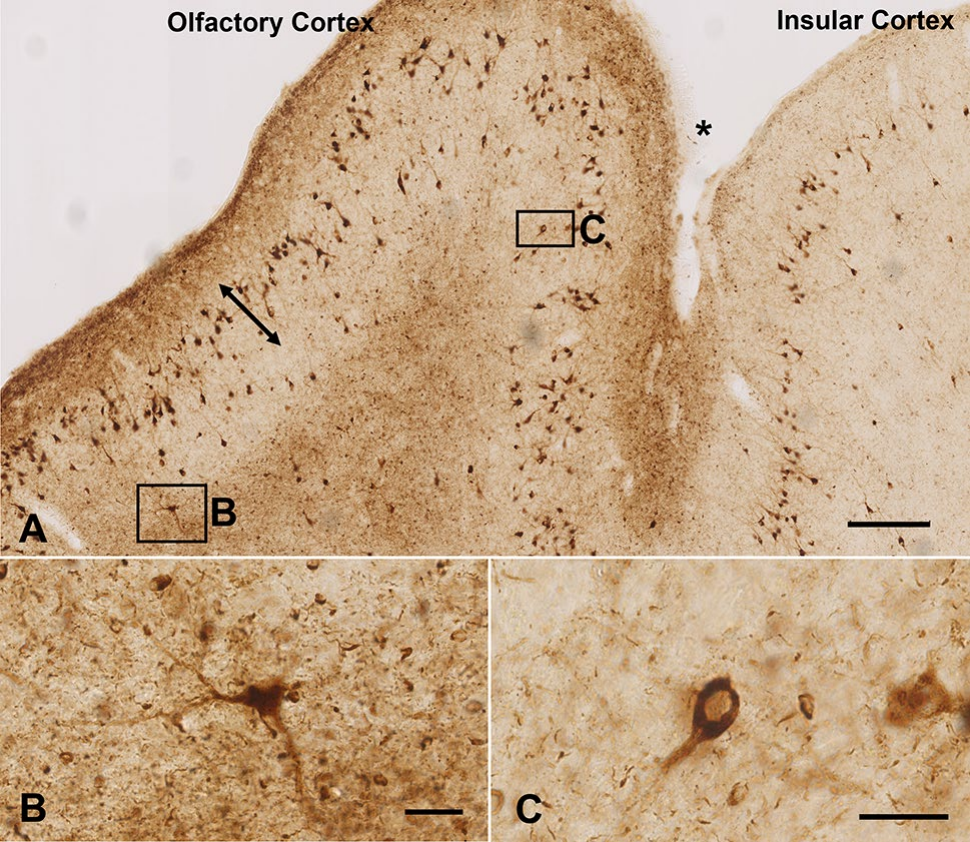
Immunohistochemistry of brain cortex of an aged sheep using the anti-phospho tau antibody AT8. **A** Numerous NFTs are visible in the insular cortex and olfactory cortex of a 21-year-old sheep (sheep #P1), primarily within layer III (arrow). The section is viewed lateromedially from top to bottom, with the rhinal sulcus labelled by *. The regions enclosed by boxes are shown in **B** and **C**. **B** An example of an AT8-positive neuron and processes in the olfactory cortex. **C** AT8-positive cytoplasmic neuron and single process. Scale bars represent 300 μm (**A**) or 50 μm (**B**, **C**)

**Fig. 2:**
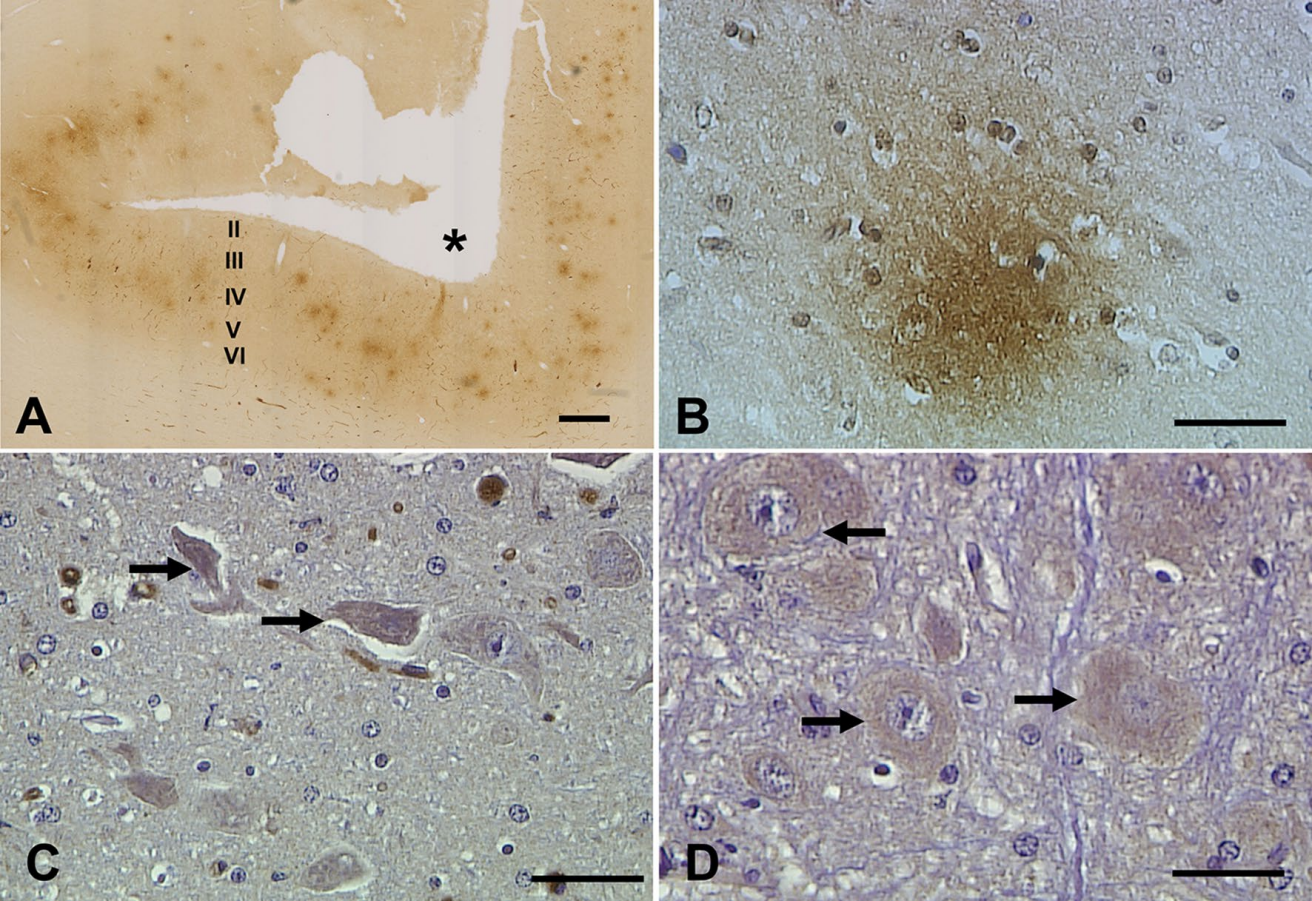
Immunohistochemistry of sheep brain using the anti-Aβ1–42 antibody ab12267. **A** Numerous diffuse Aβ plaques are visible within the parietal cortex of a sheep 16 years of age (sheep #P2). The section is viewed lateromedially from top to bottom, with the suprasylvian sulcus labelled with *. The positions of cortical layers II-VI are estimated. **B** An example of a diffuse Aβ plaque in the entorhinal cortex of a sheep > 5 years of age (sheep #5). **C**, **D** An example of positive intracellular cytoplasmic labelling of large neurons (arrows) in the pons (pontine nuclei ventrolateral and ventromedial to the pyramidal tract) of a sheep > 5 years old (sheep #6). The section has been counterstained so nuclei are stained blue. Scale bars represent 500 μm (**A**) or 50 µm (**B**–**D**)

Immunohistochemistry using the anti-phospho-tau antibody AT8 identified numerous mature NFTs throughout the neocortex of the 21-year-old sheep (P1). The NFTs were particularly concentrated within layer III of the insular cortex (claustrocortex), and the piriform cortex of the olfactory cortex (Fig. 1). No significant AD-like Aβ pathology was identified in this sheep using the Aβ1–42 antibody. In contrast, the same antibody revealed numerous diffuse, but not dense cored plaques in the parietal cortex of the 16-year-old sheep (P2) (Fig. 2A). A small number of AT8-positive neurons were also detected in the olfactory cortex and parietal cortex of the 16-year-old sheep (see Online Resource 2; Figure S1).

### β-Amyloid immunohistochemistry

#### Neuronal tissue

Immunohistochemistry using the anti-Aβ1–42 antibody, ab12267, detected a small number of diffuse Aβ deposits in the entorhinal and temporal cortex of one individual > 5 years of age (sheep #5; Table 2; Fig. 2B). No diffuse Aβ deposits were detected in 1–2-year-old sheep, and no dense-cored plaques were detected in any of the brains examined. However, this antibody labelled large neurons in the pons (pontine nuclei ventrolateral and ventromedial to the pyramidal tract) of all 1–2 and > 5-year-old sheep (for example Fig. 2C, D).

**Table 2:** Immunohistochemical detection of Aβ and phosphorylated tau in 1–2 and > 5 year old sheep

Age range (animal identifier)	Diffuse Aβ plaques (ab12267)	Vascular Aβ labelling (ab12267)	Vascular Aβ labelling (mOC64)	Hippocampal CA3 region (mOC64)	Hippocampal CA3 region (S396)	Pre-tangles (AT8)
> 5 (1–10)	+ (5)	+ (2, 5, 7, 8, 9, 10)	++ all	++ all	+ (2, 3, 9) ++ (1, 4, 5, 6, 7, 8, 10)	+ (1, 4, 6)
1–2 (11–20)	−	+ (11, 12, 18)	+ (13, 16, 17, 18, 20) ++ (11, 12, 14, 15,19)	+ (11, 13, 14, 15, 16, 17, 18, 19)	+ (12, 14, 15, 17, 19) ++ (11, 13, 16, 18, 19)	−

+; partial immunopositivity (observed in a small number of cells), ++; abundant immunopositivity, −; no immunopositive labelling, brackets indicate in which individual animal (1–10; animals > 5 years old, 11–20; animals 1–2 years old) immunopositivity was identified

**Fig. 3:**
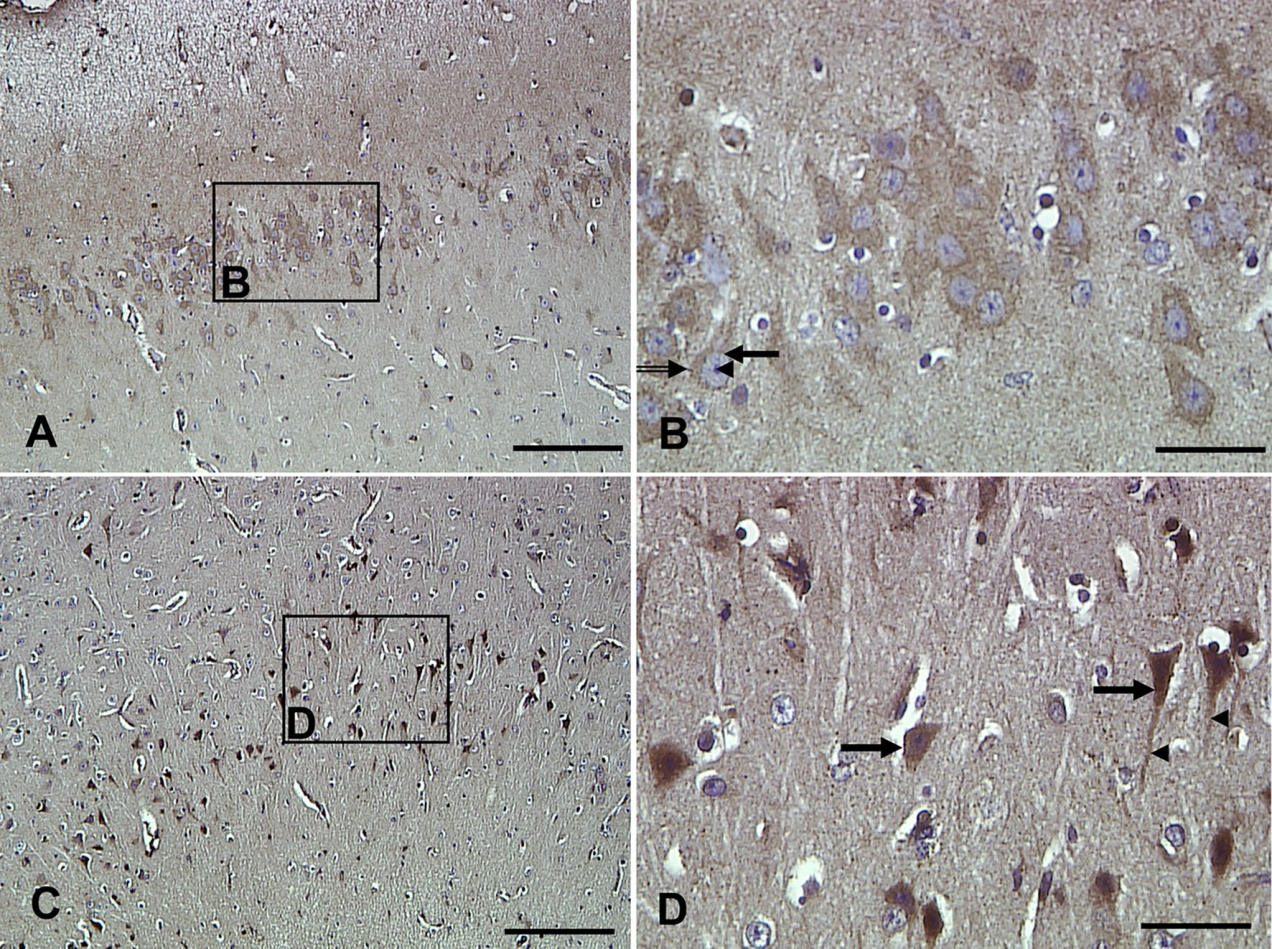
Immunohistochemistry of sheep brain using the anti-Aβ1–42 antibody mOC64. **A** Intracellular cytoplasmic staining of neurons is clearly visible as brown deposit within the entorhinal cortex (layers II and III) of a sheep > 5 years of age (sheep #3). The region enclosed by the box is shown in B. A negative control section where the primary antibody has been omitted is available within Online Resource 3 (Figure S3). **B** The neuronal nuclei (nucleolus: arrowhead, nucleus: solid arrow) are clearly defined but non-labelled, as labelling is confined to the cytoplasm (open arrow). **C** Intracellular cytoplasmic labelling of pyramidal neurons (layer V) within the entorhinal cortex of a sheep > 5 years old (sheep #5). The region enclosed by the box is shown in **D**. **D** Immunopositive staining is present in the cytoplasm and nuclei (arrows), and neurites (arrow heads). The section has been counterstained so nuclei are stained blue. Scale bars represent 200 µm (**A**, **C**) or 50 µm (**B**, **D**)

Immunohistochemistry using the anti-Aβ1–42 antibody, mOC64, labelled neurons in layers II and III of the entorhinal cortex (Fig. 3A, B), and layers III and V of the entorhinal and parietal cortices (Fig. 3C, D) of all individuals > 5 years of age. The cell bodies of the stratum pyramidale and neuronal processes of the substratum radiatum within the CA3 region of the hippocampus were also clearly labelled in all sheep > 5 years old (Fig. 6C). These cells were also labelled in 8 out of 10 1–2-year-old sheep (for details of individual sheep see Table 2).

Human AD brain samples were used as antibody control tissue. Both the anti-Aβ1–42 antibodies, ab12267 and mOC64 labelled Aβ plaques with minimal background signal present (Online Resource 3; Figure S2).

#### Vascular tissue

**Fig. 4:**
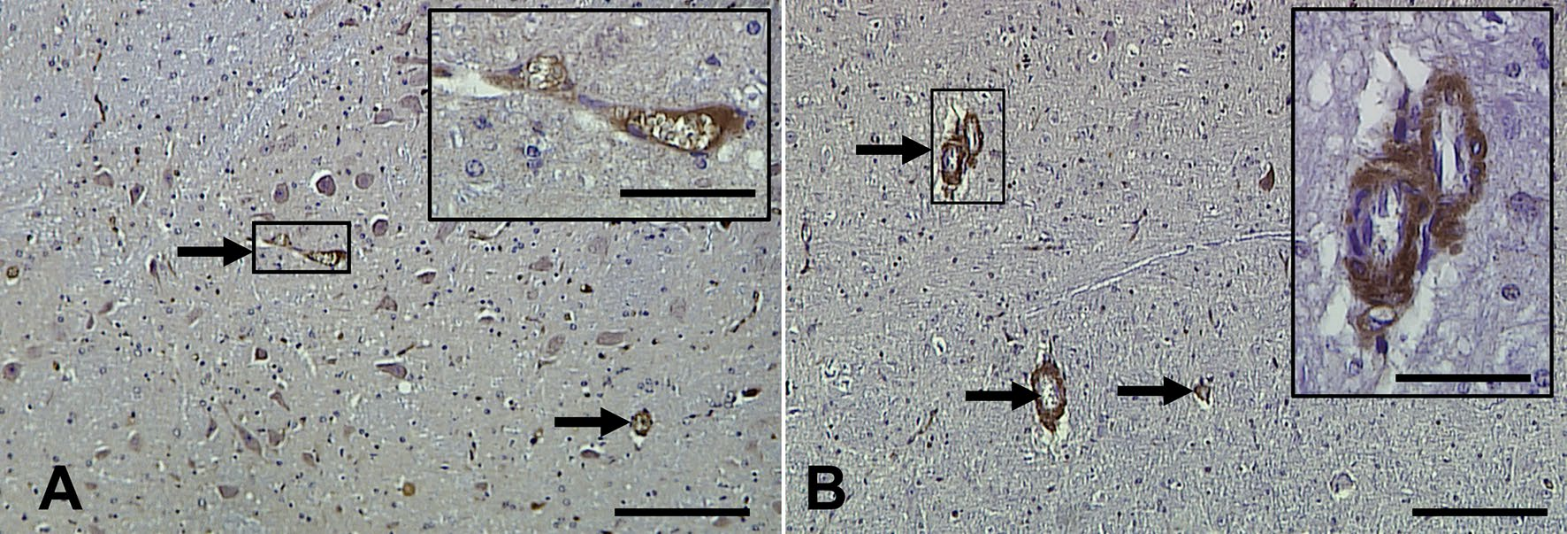
Immunohistochemistry of sheep brain (pons) using anti-Aβ1–42 antibodies. **A**, **A’** An example of ab12267 antibody labelling of small vessels (arrows) in a sheep > 5 years of age (sheep #10). **B**, **B’** An example of mOC64 antibody labelling of small vessels (arrows) in a sheep > 5 years of age (sheep #6). The section has been counterstained so nuclei are stained blue. Scale bars represent 200 µm (**A**, **B**) or 50 µm (**A’**, **B’**)

Many small arterial and capillary walls within the pons of both 1–2 and > 5-year-old sheep were positively labelled using the ab12267 antibody (for example Fig. 4). Immunopositivity was not always confined to vessel walls, and often extended into the vessel lumen. Arterial and capillary walls within the hippocampus, entorhinal cortex, parietal cortex, and pons of 1–2 and > 5-year-old sheep were also labelled using the mOC64 antibody. Vascular labelling with this antibody was confined to the vessel walls and within the tunica media.

### Tau immunohistochemistry

**Fig. 5:**
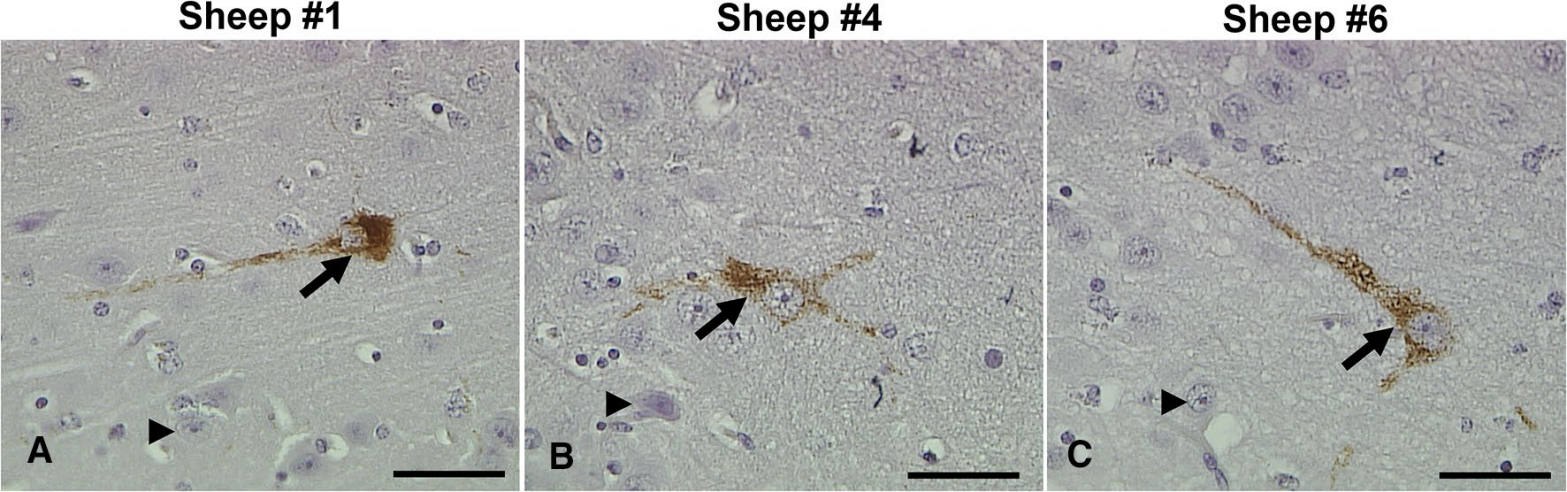
Pre-neurofibrillary tangles identified using the anti-phospho-tau antibody AT8. **A** An example of a pre-neurofibrillary tangle (arrow) where the soma is homogenously immunoreactive and the nuclei exhibit speckled staining. **B**, **C** Two examples of pre-neurofibrillary tangles (arrows) in the cell soma with nuclei that are clearly defined but unlabelled and neuritic compartments that appear speckled (arrows). Examples of non-labelled neurons are indicated in each panel (arrowheads). Scale bars represent 50 µm (**A**–**C**). The section has been counterstained so nuclei are stained blue

Immunohistochemistry using the anti-phospho-tau antibody, AT8, labelled pre-tangles within the entorhinal cortex (layer II) of three sheep > 5 years of age (sheep #1, #4 and #6) (Fig. 5). No mature NFTs were identified using the AT8 antibody in any of the brain regions of 1–2-year-old or > 5-year-old sheep.

**Fig. 6:**
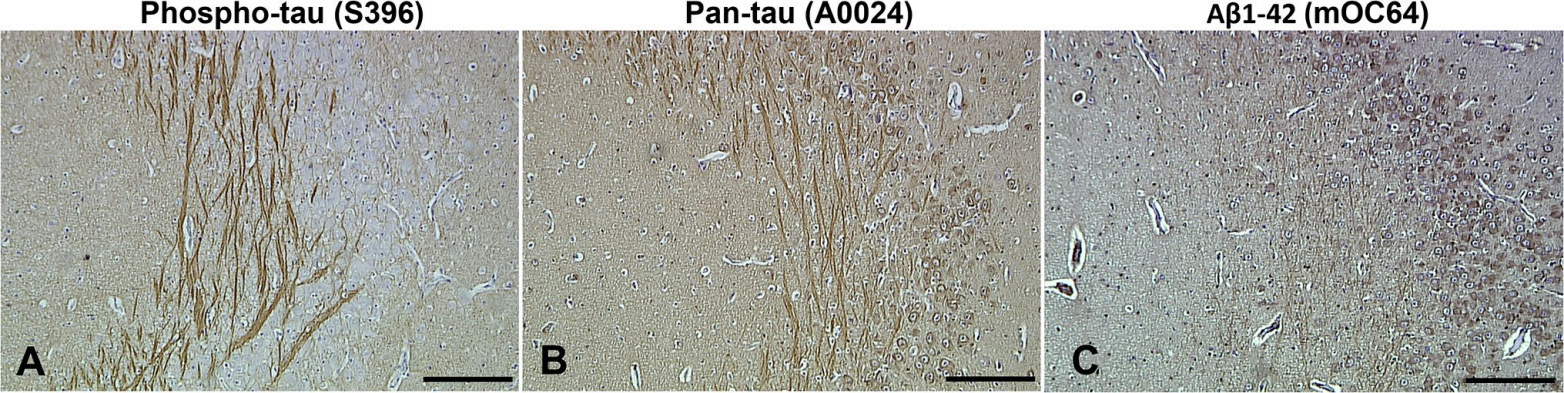
Immunohistochemistry of the CA3 region of sheep hippocampus. **A** Labelling of neuronal processes within the substratum radiatum of the CA3 region of the hippocampus in a 1–2-year-old sheep is seen after immunostaining using the anti-phospho-tau S396 antibody (sheep #16). **B** Labelling of both soma and neuronal processes in the CA3 region of the hippocampus in a 1–2-year-old sheep using the anti-pan-tau antibody DAKO0024 (sheep #16). **C** Immunolabelling of the soma and neuronal processes using the Aβ1–42 antibody, mOC64 within the CA3 region of the hippocampus of a sheep > 5 years of age (sheep #6). Scale bars represent 200 µm (**A**–**C**)

Immunohistochemistry using the anti-phospho-tau antibody, S396, resulted in a distinct pattern of immunopositivity, limited to the fibres coursing within of the substratum radiatum of the CA3 region of the hippocampus (Fig. 6A). In contrast, staining using the pan-tau antibody (A0024) revealed both fibre and neuronal positivity in CA3 (Fig. 6B), similar to that seen using the Aβ1–42 mOC64 antibody (Fig. 6C). No labelling of the cell bodies occurred when tissue was stained with the S396 antibody. This pattern of staining was clearly demonstrated in 7 out of 10 sheep aged > 5 years, and 5 out of 10 sheep aged 1–2 years. The remaining sheep exhibited partial labelling of this region that was restricted to only a few neurites (Table 2).

Both the anti-phospho-tau antibodies, AT8 and S396, labelled NFTs in human AD tissue, that was used as a positive control, with minimal background signal present (Online Resource 3; Figure S2). A schematic drawing (Online Resource 4; Figure S4) summarises the AD like-pathology identified in the sheep included in this study.

### Atypical AT8 labelling

**Fig. 7:**
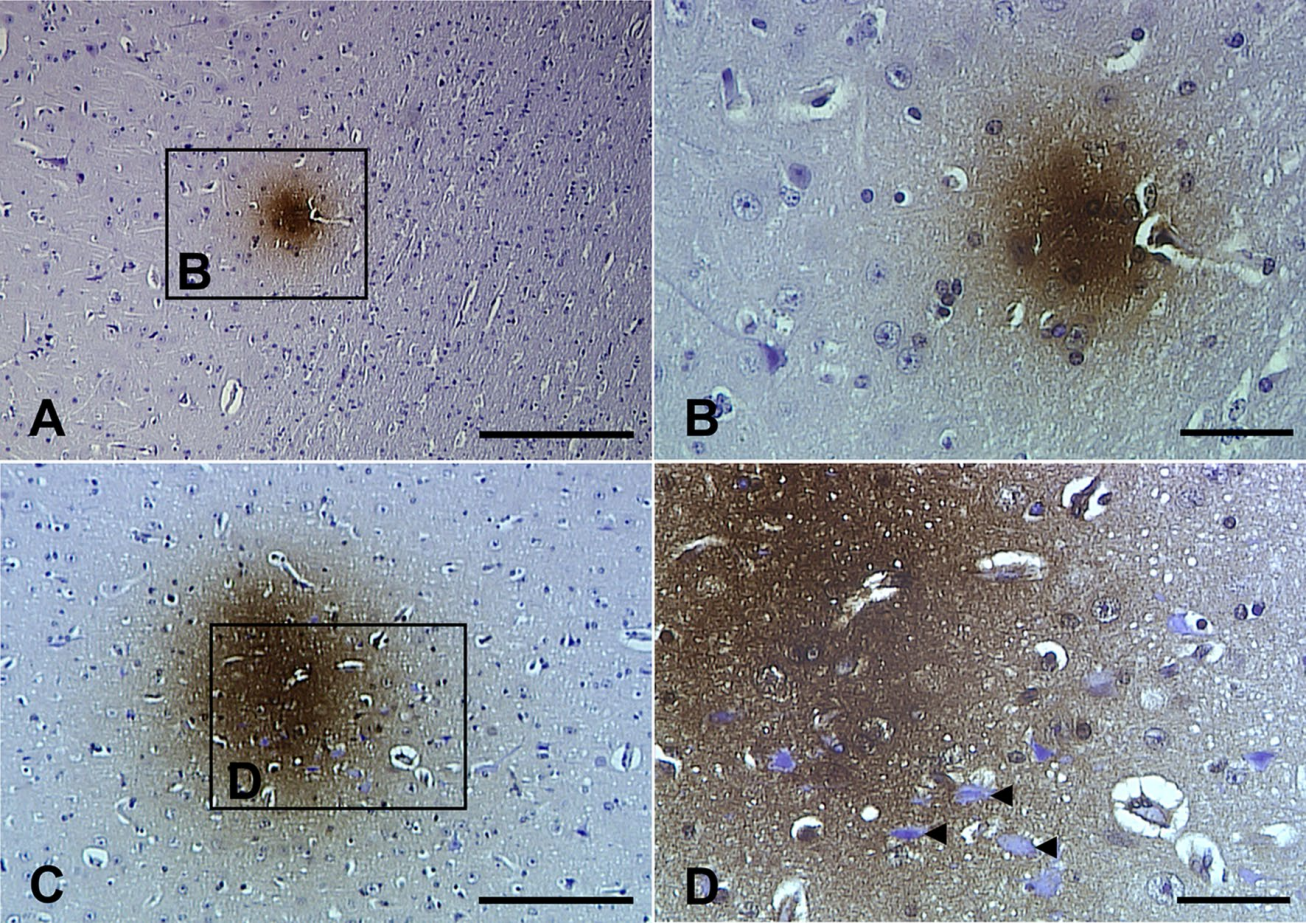
Immunohistochemistry using the anti-phospho-tau antibody, AT8, identified diffuse plaque-like regions not typical of AD tau pathology. **A**, **B** An example of a small discrete positive region within the parietal cortex, with no other staining present in the surrounding tissue (sheep #4). The region enclosed by the box in **A** is shown in **B**. **C**, **D** An example of a relatively large, intensely stained region within the parietal cortex that exhibits intra-cellular labelling, extracellular labelling, and non-labelled neurons (arrowheads) (sheep #5). The region enclosed by the box in **C** is shown in **D**. The section has been counterstained so nuclei are stained blue. Scale bars represent 200 µm (**A**, **C**) or 50 µm (**B**, **D**)

Immunohistochemistry using the anti-phospho-tau antibody, AT8, resulted in a number of diffuse plaque-like immuno-positive regions throughout the parietal cortex and pons of both 1–2- and > 5-year-old sheep. The hippocampus and entorhinal cortex of sheep > 5 years old also exhibited small numbers of these diffuse plaque-like positive areas, though none were identified within the same brain regions of 1–2-year-old sheep. These positively labelled areas did not contain typical tau pathology such as neuropil threads or NFTs. Rather they appeared as discrete amorphic structures, more closely resembling diffuse Aβ deposits, which appeared as small diffuse or large dense areas of AT8 immunostaining (Fig. 7). However, these structures were not positively labelled using Aβ antibodies or the S396 antibody and did not resemble tau pathology normally associated with dense-cored plaques. Given that all control slides were devoid of any non-specific immunoreactivity, non-specific artefacts were ruled out.

#### Atypical tau quantitation

**Fig. 8:**
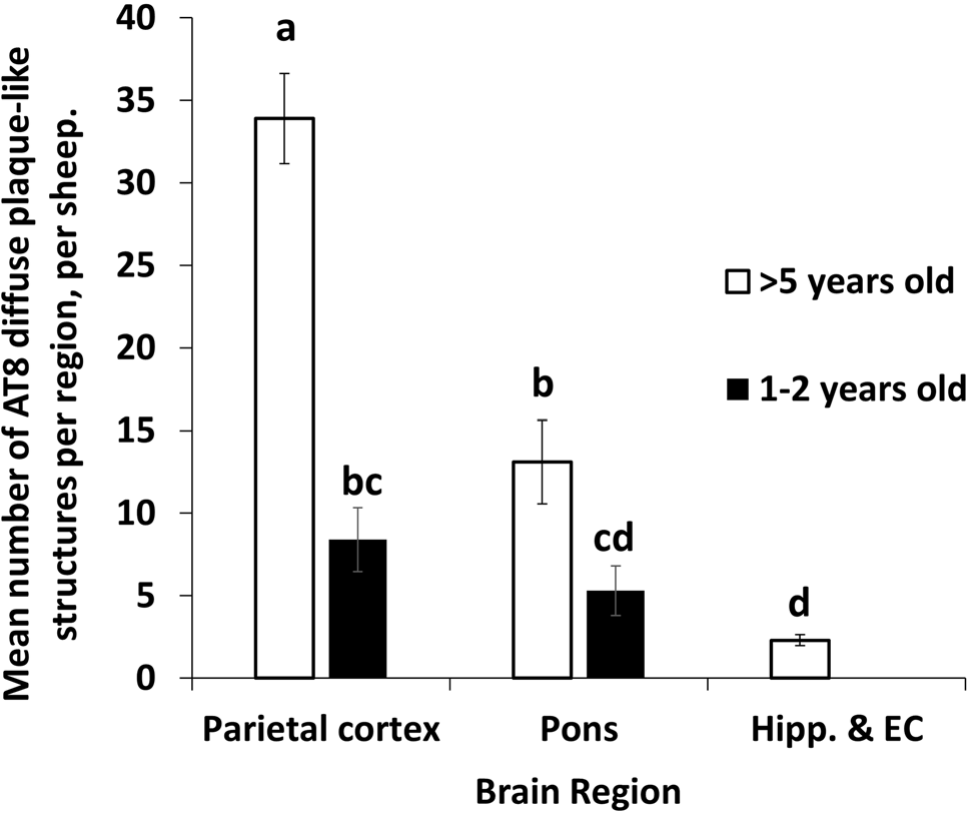
Differences in the average number of AT8 positive diffuse plaque-like regions between different brain regions and age groups of sheep. Graphical data are presented as mean ± SEM. Different letters (^a−d^) indicate significant differences (*p* < 0.05), shared letters indicate no significant differences. There was a significant interaction (*p* < 0.001) between age and brain region on the number of AT8-positive regions identified. (PC; parietal cortex, Hipp & EC; hippocampus and entorhinal cortex)

A two-way ANOVA was undertaken to test the effect of age, brain region, and also the interaction between age and brain region on the quantity of AT8-positive areas identified. Results showed that there was a significant interaction between age and brain region on the number of AT8-positive regions identified (*F* (2,54) = 22.04, *p* < 0.001). Tukey HSD post hoc tests were conducted. For sheep > 5 years of age, the number of positive AT8 areas identified in the parietal cortex was significantly greater than in the pons (*p* < 0.001) and hippocampus (*p* < 0.001). The number of AT8-positive regions identified in the pons was also significantly greater than in the hippocampus (*p* = 0.001). For sheep 1–2 years of age, the number of positive AT8 areas identified in the parietal cortex was significantly greater than in the hippocampus (*p* = 0.02). No significant differences in the number of AT8-positive areas between the parietal cortex and pons, or pons and hippocampus were detected. Additionally, the number of AT8-positive regions identified in the parietal cortex and pons of sheep > 5 years of age was significantly greater than for sheep 1–2 years of age (*p* < 0.001 and *p* = 0.042, respectively). No significant difference for the hippocampus between age groups was identified. Therefore, although the number of AT8-positive areas identified was greater for sheep that are > 5 years of age, compared to sheep that are 1–2 years of age, the difference in the number of areas identified between these two groups varied between brain regions (Fig. 8).

### Expression of tau isoforms

**Fig. 9:**
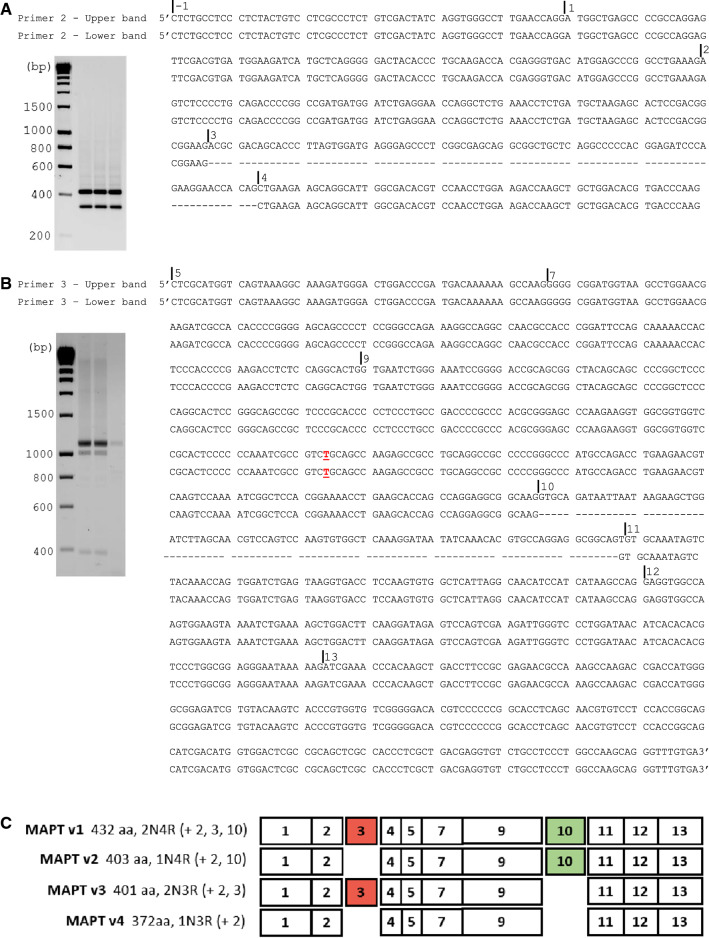
PCR cloning sequence analysis of ovine tau transcripts. **A** Primer pair 2 spanning exons-1–5, confirms the splicing of exon 3. **B** Primer pair 3 spanning exons 5–13, confirms the splicing of exon 10. Underlined nucleotide bases highlighted in red identify where nucleotides differ from those of the NCBI Predicted *Ovis aries* MAPT transcript, variant X1, XM_027974371.1. (bp; base pairs). C; The translated exons of the four central nervous system ovine MAPT isoforms identified in this study. The four transcripts, with 3(3R) and 4(4R) microtubule binding repeat regions result from the alternative splicing of exons 3 and 10

**Fig. 10:**
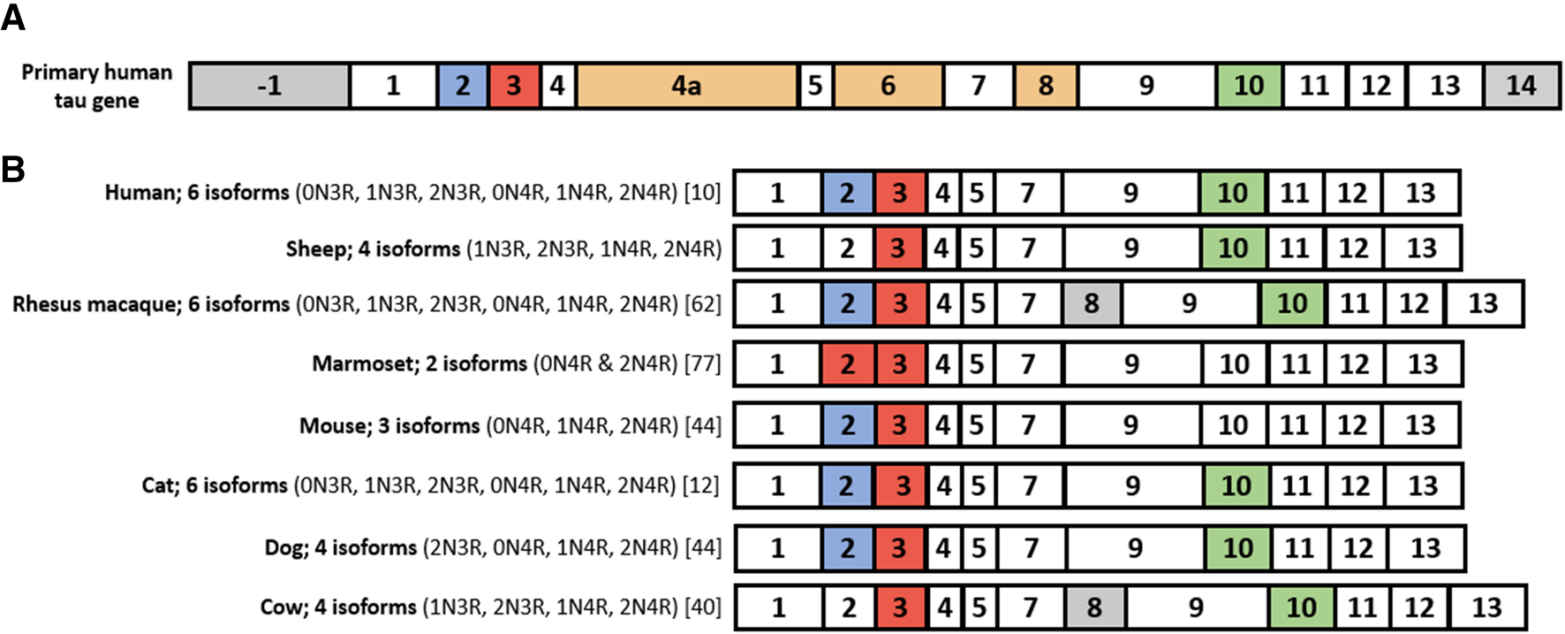
Diagrammatic representation of adult tau isoform expression in different species. Box sizes are representative of exon length. Grey boxes indicate exons that are transcribed but not translated. White boxes indicate constitutive exons that are translated. **A** The complete human tau gene consists of 16 exons. Exon-1 is part of the promotor and is transcribed but not translated. Exons 4a, 6 and 8 are specific to peripheral tau and are not transcribed in the brain. Exon 14 is transcribed but not translated. **B** A comparison of CNS tau transcripts between humans and different animal species. Exons-1 and 14 are transcribed but not translated and have been omitted from the diagram. Coloured boxes (exons 2, 3 and 10) indicate exons that are alternatively spliced. In humans, exons 2, 3 and 10 are alternatively spliced, yielding six possible isoforms. Exon splicing and tau isoform expression differs between species. Note that exon 3 does not appear without exon 2. Note that exon 8 has been identified in some bovine and rhesus macaque brain tissue transcripts, although there is no evidence it is translated

To confirm the splice variants of ovine tau, tau transcripts from one sheep (sheep #1) were examined using plasmid PCR cloning. Samples from four sheep (sheep # 1, 2, 3, 4) were also analysed using mass spectrometry. Primer pair 1 which spanned exons-1–12 resulted in the amplification of multiple cDNA products between approximately 800 and 1300 base pairs in length. Following the successful ligation of these products into the plasmid vector, and subsequent transformation into competent cells, 15 complete tau cDNA clone products were sequenced. Sequence analysis showed that while exon 2 was present in all transcripts, alternative splicing of exons 3 and 10 occurred, resulting in the expression of four distinct tau isoforms, identified as 2N4R (+ 2, 3, 10), 2N3R (+ 2, 3), 1N4R (+ 2, 10) and 1N3R (+ 2) tau isoforms (Fig. 9). The splicing of exons 3 and 10 was confirmed using primer pairs 2 and 3. Primer pair 2, which spans exons-1–5 resulted in the amplification of two distinct cDNA bands, between approximately 300 and 500 base pairs in length. The sequencing of these bands confirmed the splicing of exon 3. Primer pair 3, which spans exons 5–13 resulted in the amplification of two distinct cDNA bands, between approximately 800 and 1100 base pairs in length. The sequencing of these bands confirmed the splicing of exon 10. No indication for the splicing of exon 2, which occurs in humans was observed following PCR, plasmid cloning or transcript sequencing. Exons 4a, 6 and 8 are not transcribed in human tau, and we found no evidence to suggest transcription of these exons in ovine tau. An NCBI BLAST alignment demonstrated that the transcribed coding regions, from exon 1 to exon 13, of the experimental tau transcripts generated in this study shared 99.9% identity with the same regions of the longest predicted reference ovine tau transcript, XM_027974371.1. One nucleotide substitution, identified within exon 9, resulted in the replacement of a cytosine included in the reference transcript with a thymine, resulting in a synonymous substitution. As a result, the longest ovine tau protein, consisting of 432 amino acids, confirmed in this study, shares 90.52% homology with the longest human CNS tau isoform (NCBI, NP_005901.2), and 90.07% homology with the longest murine CNS tau isoform (NCBI, NP_001033698.1) (Online Resource 5; Figure S5). Mass spectrometric analysis identified peptides translated from each of the exons identified during the PCR cloning of cDNA, providing further evidence for the existence of four MAPT splice variants in the adult ovine cerebral cortex (Online Resource 1; table S2). A comparison of the tau variants expressed in the adult brains of a range of species is included in Fig. 10.

### Phosphorylation of tau serine396

**Fig. 11:**
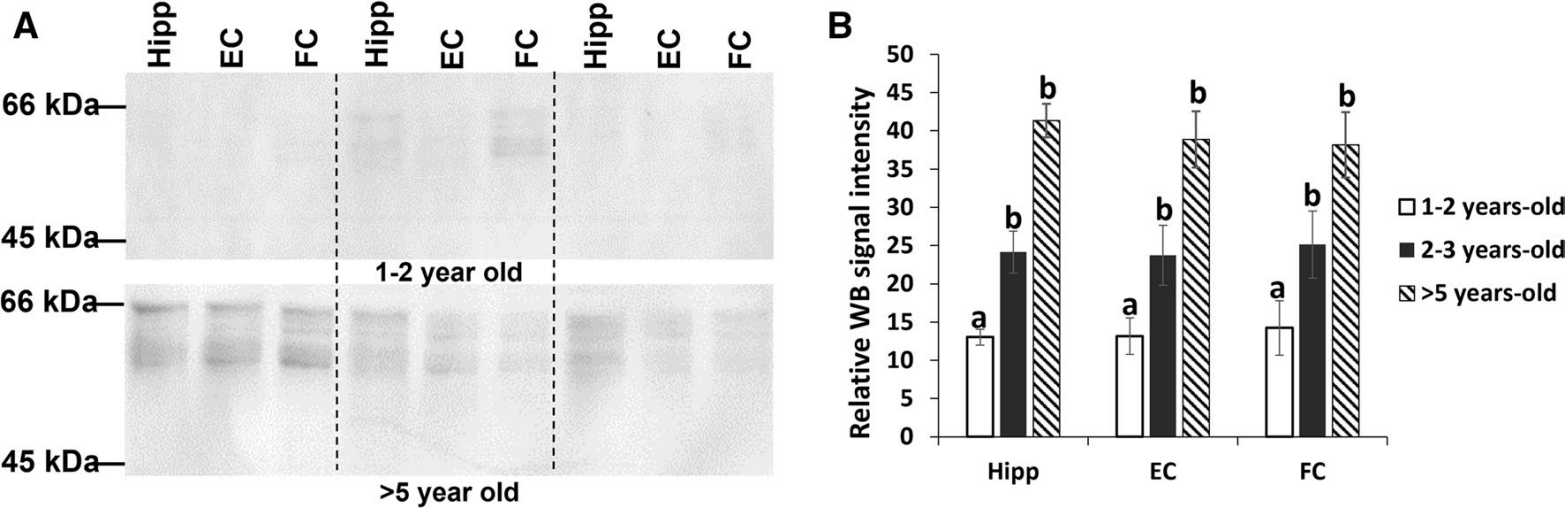
Western blot analysis using anti-phospho-tau antibody, S396. **A** Representative western blot of three brain regions from three sheep > 5 years of age (upper panel; sheep #1, #5 and #7), and three sheep 1–2 years of age (lower panel; sheep #15, #16 and #17). **B** Average relative western blot signal intensities of all brain samples analysed using western blot. Data are presented as mean ± SEM. Different letters ^(ab)^ indicate significant differences. There was no statistically significant difference in mean intensity between brain regions, but there were statistically significant differences (*p* < 0.001) between age groups (Hipp; hippocampus, EC; entorhinal cortex, FC; frontal cortex)

Tau phosphorylation at residue serine396 was examined using western blotting. Tau phosphorylated at this epitope was detected in soluble samples from the hippocampus, entorhinal cortex and frontal cortex of all sheep > 5 years of age, in 7 out of 10 sheep 2–3 years of age, and 2 out of 10 sheep 1–2 years of age (sheep #16 and #17). When the relative intensities of the western blots were compared (Fig. 11), there was no statistically significant difference in mean intensity between brain regions (*F* (2,81) = 0.259, *p* = 0.772), but there were statistically significant differences between age groups (*F* (2,81) = 18.26, *p* < 0.001). There was no statistically significant interaction of these two factors on mean intensity (*F* (4,81) = 0.129, *p* = 0.971). Post hoc analysis indicated that signal intensity of tau abundance in sheep > 5 years of age was significantly greater than the signal intensity of sheep 1–2 years of age (*p* < 0.001). The signal intensity of sheep 2–3 years of age was also significantly greater than that of sheep aged 1–2 years old (*p* < 0.001). However, there were no statistically significant differences in signal intensity when sheep 2–3 and > 5 years old were compared directly (*p* = 0.195).

## Discussion

### Aβ pathology

Previous studies have reported that both Aβ and tau pathology occurs naturally in sheep brain, which support these findings [64, 74]. Our pilot study using two very old sheep identified numerous mature NFTs and diffuse Aβ deposits. We extended these findings by characterising AD-like Aβ and tau pathology in sheep sourced from the commercial sheep population. We detected a small number of diffuse Aβ deposits in the entorhinal cortex of only one individual aged > 5 years but found intraneuronal Aβ1–42 labelling using the mOC64 antibody in all sheep > 5 years of age. The presence of intraneuronal oligomeric Aβ species is considered an early aetiological event that precedes extracellular plaque and NFT formation and has been described extensively in humans with AD [19, 33, 34, 59, 70, 91, 98], and in transgenic mouse models of AD [52, 68, 69]. Intraneuronal oligomeric Aβ has also been described in cases of spontaneous AD-like pathology in cats [12], dogs [17] and dolphins [88].

Vascular Aβ deposition was detected throughout the brains of 1–2 and > 5-year-old sheep. The presence of vascular Aβ labelling in the absence of parenchymal Aβ deposition is particularly interesting as it has been hypothesised that both red blood cell and cerebral vascular damage may be key processes that contribute to the development of Aβ plaques [53, 83]. It is not clear whether these processes lead to increased parenchymal Aβ deposition due to cellular hypometabolism or increase the influx of Aβ enriched proteins from blood derived cells. However, it has been suggested that CAA may represent an intermediate stage in the process of Aβ plaque formation [25, 42, 53, 89].

Aβ deposition and NFTs have previously been identified in 8-year-old sheep using the monoclonal mouse antibody, clone 4G8 [74]. Those Aβ deposits were described as large dense structures, occurring throughout all cortical regions, including the hippocampus. Numerous small areas of staining, the size of a single neuronal nuclei, were also described. Aβ deposits consistent with those described by Reid et al. [74] were not identified in this study. The absence of large Aβ deposits may be due to the younger age of the sheep we examined. The absence of small deposits, however, is more likely to reflect the use of a different antibody. Reid et al. [74] used the clone 4G8, that recognises the Aβ epitope between amino acids 18 and 22 and is unable to distinguish Aβ from APP and other Aβ-containing APP fragments [2, 34]. As a result, the amount of Aβ deposition reported previously may have been overestimated.

### Tau pathology and phosphorylation

In the current study, three AT8-positive pre-neurofibrillary tangles [5] were identified in sheep > 5 years of age, and numerous mature NFTs were detected in sheep 16 and 21 years of age. In humans, the differential phosphorylation of tau residues occurs in a temporal pattern correlated with disease progression [93]. Phosphorylation of residue serine396 is generally considered to be an early pathological event, associated with tau oligomerization [31, 54, 60, 73, 97]. Thus, the S396 antibody is commonly used to study early tau hyperphosphorylation in a range of species and AD models [1, 29]. In contrast, the phosphorylation of residues serine202 and threonine205, detected by the AT8 antibody, occurs in the latter stages of tau pathology [32]. Although it is likely that the absence of mature NFTs in the stock sheep reflects the relatively young age of the sheep, the presence of pre-tangles is indicative of early-stage tau pathology in sheep as young as 5–8 years of age [74]. As the preliminary study of a 21-year-old sheep detected numerous AT8-positive mature NFTs, it is likely that older sheep display advanced stage tau pathology as observed in AD, and that the pre-tangle pathology observed in younger sheep may eventually progress to this advanced stage. The variation in spontaneous tau and Aβ pathology observed between the very aged sheep in the pilot study also reflects the significant variability of tau and Aβ pathology observed in aged humans, further pointing to the sheep as a relevant animal model.

Phosphorylation of the serine396 residue was also investigated. We found phosphorylated S396 immunoreactivity was present in some sheep 2 years of age and that phosphorylation increased with age. While the observation of this pathology at such an early age may be surprising, it is consistent with human data where it has been shown that abnormal tau changes occur in the brain as early as childhood or puberty [8, 9]. Recent evidence also suggests that neurons undergo a dying back pattern of degeneration, where tau pathology begins in the axonal compartment before progressing to the somatodendritic compartment [14, 51]. The differential patterns of immunostaining within the CA3 region of the hippocampus observed using different anti-Aβ1–42 and anti-tau antibodies in this study may indicate early hippocampal intraneuronal Aβ deposition, accompanied by early-stage axonal tau phosphorylation. The presence of mOC64 and S396 immunopositivity may also indicate that these changes occur in sheep as young as approximately 2 years of age. The pattern of S396 labelling within the CA3 region of the hippocampus is remarkably similar to labelling detected using the same antibody in the hippocampus of cows with idiopathic brainstem neuronal chromatolysis (IBNC) [48]. While the exact aetiological cause of IBNC is unknown, studies have indicated that it is a complex proteinopathy characterised by significant hyperphosphorylated tau with associated secondary accumulations of alpha-synuclein and ubiquitin, without associated NFT formation or amyloid deposition [48]. Interestingly, while cases of IBNC are predominantly reported in cows > 6 years old, the youngest recorded case was in a cow just 4 years old. Therefore, further research into tau phosphorylation and tauopathy in sheep and large ruminants would be beneficial to determine if these two pathologies are related.

The current study identified diffuse plaque-like tau-positive deposits that were not characteristic of AD-like tau pathology. We found no background staining in the tissue surrounding these focal regions. All control slides were also negative, and the pattern of staining with the AT8 antibody on both AD-positive and AD-negative human control tissue revealed staining only of the AD brain. Given that the regional and age-dependent distribution of these AT8-positive elements is consistent with a progressive pathogenesis, it is possible that the AT8 antibody in this instance was accurately labelling phosphorylated tau. However, equally, while the use of the AT8 antibody is renowned for achieving specific phospho-tau labelling with little or no background signal [7, 30], this pattern of atypical staining may have resulted from non-specific antibody staining, tissue treatment artefacts, or cross reactivity with another species of tau. Further investigations into possible sources of this atypical AT8 labelling in sheep are necessary.

### Tau isoform expression

In humans, all six of the tau isoforms expressed in the brain become hyper-phosphorylated and involved in tau pathology. MAPT splicing and tau isoform expression, however, differs between species, affecting the subsequent formation of tau pathology [40, 84, 92]. This study confirmed the expression of the four distinct ovine tau isoforms described by Janke et al. [47], formed from the splicing of exons 3 and 10. This pattern of ovine tau expression is consistent with the phylogenetic expression of tau in other ruminants [43, 47]. While exon 8 has previously been identified in transcripts from the rhesus macaque and the cow [66] the ovine tau transcripts generated in this study confirmed that exons 4a, 6 and 8 are not transcribed in sheep (for comparisons of tau expression between species, see Fig. 10). Janke et al. [47] also described two additional isoforms in sheep, interpreted as the two 0N tau isoforms, consistent with the two shortest tau isoforms expressed in humans. However, we found no evidence for the expression of these isoforms, resulting from the alternative splicing of exon 2. The confirmation that sheep express the 3R and 4R tau isoforms that are expressed in humans is an important step in assessing of their suitability as an AD model, particularly given that mice naturally express only 4R tau [3]. Possessing both 3R and 4R endogenous tau isoforms confers considerable potential to the sheep as an AD model with a highly translatable value.

The variations in the tau N-terminal domains of different species have been cited as reasons why tau pathology differs between species [3]. The human N-terminal domain contains an eleven amino acid motif (residues 17–28) not present within the N-terminal domain of murine tau [40]. This additional peptide sequence is thought to affect the intramolecular interactions between the N- and C-terminals, and the microtubule-binding domains of tau, increasing the likelihood of normal tau undergoing pathological conformational changes [3, 40]. The lack of this N eleven amino acid motif in murine tau has been suggested as one reason why mice do not naturally develop tau pathology [40]. Additionally, within the first 190N-terminal amino acids, human tau contains 33 serine, threonine, and tyrosine residues. Murine tau shares 22 of these residues. Many of the residues, found in humans (but lacking in mice) are phosphorylated in AD, or are phosphorylated by kinases that exhibit dysregulation in AD [37]. However, an alignment of the human, murine and ovine tau proteins show that sheep lack the eleven amino acid sequence, and many of the same phosphorylation sites as murine tau, even though they naturally develop tau pathology [7, 64, 65, 74]. We speculate that while the presence of the 11 amino acid motif and the phosphorylation of these residues may accelerate pathological tau formation in humans, their involvement is not essential for the development of PHF tau.

Proline-rich regions facilitate the phosphorylation of serine and threonine residues via kinases thought to have key roles in the pathogenesis of AD [44]. There are seven distinct variations between the human and ovine proline-rich regions of tau that may be implicated in tau hyperphosphorylation in the sheep. For example, proline176, alanine178 and proline182 in human tau are substituted with threonine, threonine and serine, respectively, in ovine tau. However, proline residues that are important for the directed phosphorylation of tyrosine residues such as proline213, proline216 and proline219 are conserved between humans and sheep [40, 56]. Additionally, each of the four KXGS motifs which can be phosphorylated by multiple kinases, and the four PGGG sequences which facilitate the formation of type II β-turns and β-hairpin structures, are conserved between humans and sheep [13, 21].

### Future work

As only three other studies have applied phosphorylation dependent antibodies to study the ovine brain, further work is required to elucidate the residue specific patterns of tau phosphorylation in sheep as they age. Although this study focused on the hippocampus and entorhinal cortex, critical regions for the development of AD pathology, future studies would benefit from analysing other cortical and subcortical structures that have been implicated in the early tau pathology of both humans and aged canines, such as the thalamus [1]. The investigation of other tau post-translational modifications, such as truncation would also be valuable as truncation has been associated with increased tau aggregation in AD [55]. A complete evaluation of other protein pathologies, markers of astrogliosis and neuroinflammation, such as those involving ubiquitin, APOE and GFAP would also be beneficial since this would provide a more complete picture of AD-related pathology in sheep [57]. Environmental factors that could increase the likelihood of sheep developing AD-like pathologies also need to be investigated. For example, both copper (to which sheep are sensitive), and rumen-protected feed ingredients such as formaldehyde are factors that have been implicated in AD pathogenesis [48, 87, 99]. They may contribute to the AD-like pathology observed in other young ruminants. Future studies will also benefit from a complete evaluation of potential heterozygous substitutions and single nucleotide polymorphisms of the ovine MAPT transcript. Finally, screening sheep for relevant alleles, such as APOEε4 may help to enhance the detection of AD-like pathological characteristics in the commercial sheep population, increasing their potential suitability of being a natural AD model [74, 94].

## Conclusion

We identified intracellular and vascular Aβ deposition in the brains of sheep as young as 2 years of age. We also identified AT8-positive pre-neurofibrillary tangles in sheep > 5 years of age. One of the early stages in the conversion of normal to pathological tau is the phosphorylation of multiple residues. We show that even by 2 years of age, some phosphorylation of the serine396 residue is present in sheep, and the relative amount of tau phosphorylated at this residue increases with advancing age. Given that these findings are consistent with early-stage AD-like pathology, sheep could be used to investigate the early biochemical and structural changes that eventually result in advanced AD-like pathology, without the complications of late-stage pathology. This would be an important step, given that the disease course of rodent transgenic AD models often progresses too rapidly for a comprehensive evaluation of this pre-clinical phase of AD aetiology to be completed [23]. Our pilot study detected numerous NFTs in a sheep 21 years of age, indicating that the development of late-stage tau pathology is possible in older sheep. This study has also confirmed that sheep express four distinct tau isoforms in their CNS, composed of three and four binding repeats, which result from the alternative splicing of exons 3 and 10. As a result, the sheep, either as a spontaneous model with genetic predisposition or, as a transgenic model, has the potential to provide a valuable model of early-stage AD-like tauopathy, reflective of that seen in humans.

## Supplementary Information

Below is the link to the electronic supplementary material.

Supplementary file1 (PDF 188 KB)

Supplementary file2 Fig. S1 Immunohistochemistry of brain cortex from a sheep 16 years of age (sheep #P2) using the anti-phospho tau antibody AT8. A; An example of an NFT where the cytoplasm and neurites remain defined. B; Examples of NFTs where the nucleus and cytoplasm are not distinguishable. Scale bars represent 50 μm (A) or 100 μm (B) (TIF 2847 KB)

Supplementary file3 Fig. S2 Immunohistochemistry of AD positive (top row) and AD negative (bottom row) human control tissue. The anti-phospho tau antibody, AT8 (A & E), the anti-phospho tau antibody, S396 (B & F), the anti-Aβ1–42, ab12267 (C & G), and the anti-Aβ1–42 mOC64 antibody (D & H). Scale bars represent 200 µm (A-H). Fig. S3 Immunohistochemical control section from the entorhinal cortex of a sheep > 5 years of age. The primary antibody (anti-Aβ1–42 mOC64, as seen in Fig. 3; A) has been omitted. Scale bar represents 200 µm (TIF 20936 KB)

Supplementary file4 Fig. S4: Schematic drawing of coronal sections of the sheep brain, representing the rostro-caudal distribution of AD-like pathologies in very old study pilot sheep (A; P1, B; P2), and stock sheep > 5 years of age (C). The left hemisphere viewed from the rostral aspect is illustrated. The approximate rostro-caudal positions of the coronal sections are illustrated in D. Solid triangles represent mature neurofibrillary tangles labelled with the anti-phospho-tau antibody AT8. Open triangles represent pre-neurofibrillary tangles and neuronal processes labelled with the anti-phospho-tau antibodies AT8 and S396. Solid circles represent diffuse plaques labelled with the anti-Aβ1–42 antibody ab12267. Open circles represent intra-neuronal labelling identified using the anti-Aβ1–42 antibody mOC64. The area enclosed by the box (B) represents the approximate location of Fig. 2; A. All gyri and sulci are labelled using the stereotaxis atlas of the ovine brain developed by Ella et al. [27]. C, caudate nucleus; CG, cingulate gyrus; EC, entorhinal cortex; F, fimbria; H, hippocampus; I, insular cortex; IC, internal capsule; LV, lateral ventricle; OC, olfactory cortex; OV, olfactory ventricle; P, putamen; RS, rhinal sulcus; SG, sylvian gyrus; SS, suprasylvian sulcus; SSG, suprasylvius gyrus (TIF 31288 KB)

Supplementary file5 (PDF 164 KB)

## Data Availability

Supplementary information accompanies this paper. Datasets analysed and presented in the current study are available from the corresponding author on request.

## Abbreviations

AD: Alzheimer’s disease
Aβ: β-Amyloid
CAA: Cerebral amyloid angiopathy
NFTs: Neurofibrillary tangles
APP: Amyloid precursor protein
PSEN1: Presenilin 1
PSEN: Presenilin 2
FTDP-17: Frontotemporal dementia with parkinsonism-17
FAD: Familial Alzhimer’s disease
TBS: Tris-buffered saline
RT: Room temperature
DTT: Dithiothreeitol
TTBS: Tween 20 tris buffered saline
BCIP/NBT: 5-Bromo-4-chloro-3-indolyl phosphate/nitro blue tetrazolium
IPTG: Isopropyl β-d-1-thiogalactopyranoside
X-GAL: 5-Bromo-4-chloro-3-indolyl β-d-galactopyranoside
X-GAL: Luria–Bertani
IBNC: Idiopathic brainstem neuronal chromatolysis
CNS: Central nervous system
MAPT: Microtubule associated protein tau
PCR: Polymerase chain reaction
ANOVA: Analysis of variance
CA3: Cornu ammonis 3
NCBI: National Centre for Biotechnology Information
PHFs: Paired helical filaments
APOE: Apolipoprotein
GFAP: Glial fibrillary acidic protein
