# Supplementary material for: Characterization of microtubule-associated protein tau isoforms and Alzheimer’s disease-like pathology in normal sheep (Ovis aries): relevance to their potential as a model of Alzheimer’s disease
Source: Cell Mol Life Sci. 2022 Oct 21;79(11):560. doi: 10.1007/s00018-022-04572-z (PMC9587068; doi:10.1007/s00018-022-04572-z)
Supplement: Supplementary file 1 — Supplementary file1 (PDF 188 KB) [file 18_2022_4572_MOESM1_ESM.pdf]

Characterization of microtubule associated protein tau isoforms and Alzheimer's disease-like pathology in normal sheep (*Ovis aries*) - relevance to their potential as a model of Alzheimer's disease.

Authors: Emma S. Davies[a], Russell M. Morphew[a], David Cutress[a], A. Jennifer Morton[b], Sebastian McBride[a]

[a] Department of Life Sciences, Aberystwyth University, Aberystwyth, Wales.

[b] Department of Physiology, Development and Neuroscience, University of Cambridge, Cambridge, UK

Correspondence: sdm@aber.ac.uk, +44 (0) 1970 621633, Institute of Biological, Environmental and Rural Sciences, Aberystwyth University, Aberystwyth, Wales.

Email: Emma S. Davies; esd5@aber.ac.uk, Russel M. Morphew; rom@aber.ac.uk, David Cutress; djc25@aber.ac.uk, A. Jennifer Morton; ajm41@cam.ac.uk, Sebastian McBride; sdm@aber.ac.uk

Pilot Experiment Methodology

A pilot study was conducted using brain tissue from two aged pet female sheep that were euthanised for humane reasons by a veterinary surgeon at the request of their owner. The owner subsequently donated their brains for this study. One sheep was a 21-year-old Derbyshire Gritstone (P1), the second was a 16-year-old Suffolk crossbred (P2). The brains were extracted from the skull and immersed in 4% paraformaldehyde for one month. All membranes covering the cortex and cerebellum were removed and the brain was cut into two hemispheres and then smaller blocks. Blocks were transferred into a cryopreservation solution (20% sucrose). The tissue was cut into 60µm sections and stored in phosphate-buffered saline containing 0.5% sodium azide until required. Immunohistochemical examination was conducted using the anti-phospho-tau antibody AT8 (Invitrogen, MN1020; 1:2500 dilution) and the anti-Aβ antibody (Abcam, 12267; 1:200 dilution). Free floating sections of tissue were incubated in primary antibody for two weeks. Non-specific binding was blocked using 4% normal goat serum and peroxidase activity was inactivated using H_2O_2. The primary antibody was visualised using an HRP-conjugated secondary antibody and 3,3'-diaminobenzidine. Sections were mounted onto glass slides and cover-slipped.

Human Tissue Sample Description

Human hippocampal tissue samples were obtained from the Medical Research Council UK Brain Bank Network (the South West Dementia Brain Bank (SWDBB)). The tissue samples were histologically evaluated by the SWDBB. One sample (BBN006.36298) had a diagnosis of severe AD (AD positive), and the other sample (BBN006.35942) was described as having no significant neuropathological abnormalities (AD negative). These tissue samples were used as human AD positive and AD negative control samples.

Table S1: Primer pairs used for the PCR amplification of cDNA. Primer pairs were designed using the NCBI Predicted *Ovis aries* MAPT transcript, variant X1, XM_027974371.1.

Pair	Forward (F) & Reverse (R) Primers	TM (°C)	GC%	Within exon	Exon 2, 3, 10	Predicted length (bp)
1	F- CTCTGCCTCCCTCTACTGTC	58.60	60.00	-1	+ + +	1103
	R- CAGGGACCCAATCTTCGACT	59.09	55.00	12	+ + -	1010
					+ - +	1016
					+ - -	923
					- - +	929
					- - -	836
2	F- CTCTGCCTCCCTCTACTGTC	58.60	60.00	-1	+ +	419
	R- GCCTTTACTGACCATGCGAG	58.99	55.00	5	+ -	332
					- -	245
3	F- CTCGCATGGTCAGTAAAGGC	58.99	55.00	5	+	1087
	R- ACCGATGAACCGATCTGTGA	58.82	50.00	14	-	994

-TM and GC % calculated using the NCBI primer design tool.

-Predicted length in base pairs (bp) is calculated using the NCBI transcript, XM_027974371.1.

Table S2: Sequences, mascot scores and exon locations (X) of tau peptides identified using liquid chromatography mass spectrometry.

		Exon													
**Peptide Sequence**	Mascot Score	1	2	3	4	4a	5	6	7	8	9	10	11	12	13
ESPLQTPADDGSEEPGSETSDAK	252	X	X	-	-	-	-	-	-	-	-	-	-	-	-
SGYSSPGSPGTPGSR	39	-	X	-	-	-	-	-	-	-	-	-	-	-	-
STPTAEAEEAGIGDTSNLEDQAAGHVTQAR	187	-	X	-	X	-	X	-	-	-	-	-	-	-	-
STPTAEDATAPLVDEGALGEQAAAQAPTEI<br>-PEGTTAEEAGIGDTSNLEDQAAGHVTQAR	169	-	X	X	X		X	-	-	-	-	-	-	-	-
DGTGPDDK	347	-	-	-	-	-	X	-	-	-	-	-	-	-	-
GAAPPGQKGQANATR	163	-	-	-	-	-	-	-	X	-	-	-	-	-	-
GADGKPGTK	300	-	-	-	-	-	-	-	X	-	-	-	-	-	-
TSPGTGESGK	252	-	-	-	-	-	-	-	X	-	X	-	-	-	-
IGSTENLK	252	-	-	-	-	-	-	-	-	-	X	-	-	-	-
TPSLPTPPTREPK	252	-	-	-	-	-	-	-	-	-	X	-	-	-	-
VAVVRTPPK	70	-	-	-	-	-	-	-	-	-	X	-	-	-	-
LQAAPGPMPDLK	88	-	-	-	-	-	-	-	-	-	X	-	-	-	-
SGYSSPGSPGTPGSR	75	-	-	-	-	-	-	-	-	-	X	-	-	-	-
SRLQAAPGPMPDLK	252	-	-	-	-	-	-	-	-	-	X	-	-	-	-
GESGKSGDR	187	-	-	-	-	-	-	-	-	-	X	-	-	-	-
LDLSNVQSK	252	-	-	-	-	-	-	-	-	-	-	X	-	-	-
KLDLSNVQSK	252	-	-	-	-	-	-	-	-	-	-	X	-	-	-
HVPGGGSVQIVYKPVDLSK	252	-	-	-	-	-	-	-	-	-	-	X	X	-	-
VQIVYKPVDLSK	295	-	-	-	-	-	-	-	-	-	-	-	X	-	-
IGSLDNITHVPGGGNK	252	-	-	-	-	-	-	-	-	-	-	-	-	X	-
HLSNVSSTGSIDMVDSPQLATLADEVSASLAK	252	-	-	-	-	-	-	-	-	-	-	-	-	-	X
TDHGAEIVYK	252	-	-	-	-	-	-	-	-	-	-	-	-	-	X
SPVVSGDTSPR	252	-	-	-	-	-	-	-	-	-	-	-	-	-	X
AKTDHHGAEIVYK	252	-	-	-	-	-	-	-	-	-	-	-	-	-	X
