# Supplementary figures and images for: Characterization of microtubule-associated protein tau isoforms and Alzheimer’s disease-like pathology in normal sheep (Ovis aries): relevance to their potential as a model of Alzheimer’s disease
Source: Cell Mol Life Sci. 2022 Oct 21;79(11):560. doi: 10.1007/s00018-022-04572-z (PMC9587068; doi:10.1007/s00018-022-04572-z)

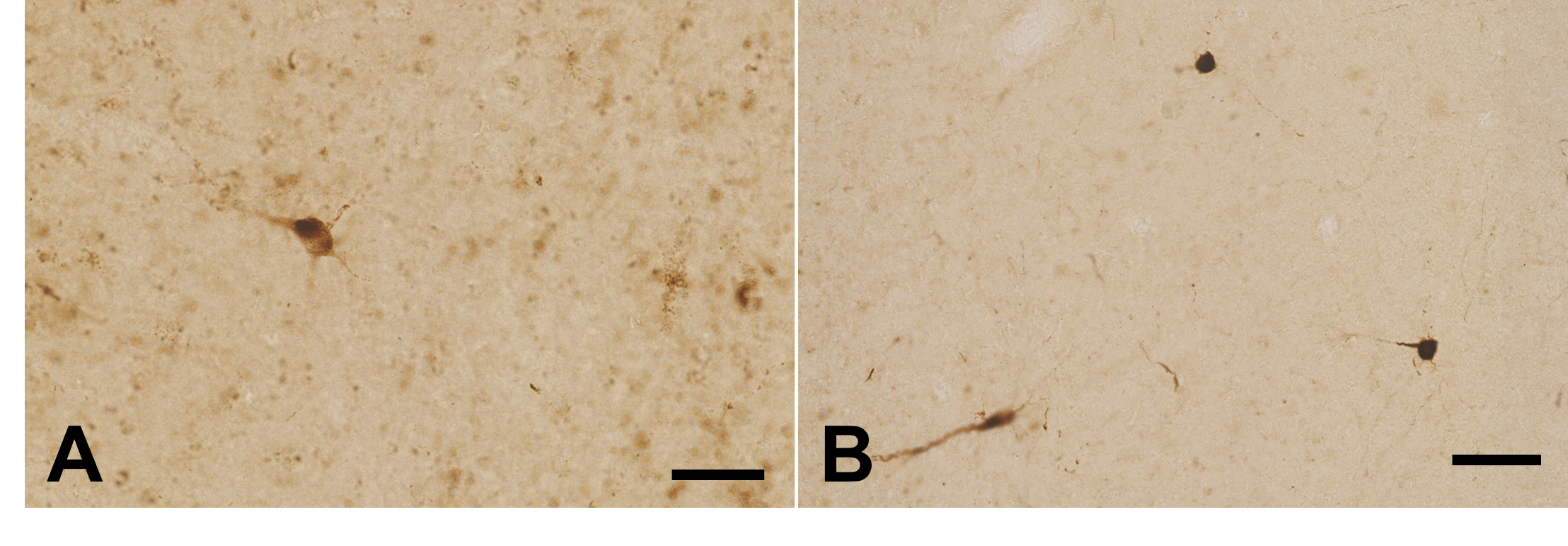

Supplement: Supplementary file 2 — Supplementary file2 Fig. S1 Immunohistochemistry of brain cortex from a sheep 16 years of age (sheep #P2) using the anti-phospho tau antibody AT8. A; An example of an NFT where the cytoplasm and neurites remain defined. B; Examples of NFTs where the nucleus and cytoplasm are not distinguishable. Scale bars represent 50 μm (A) or 100 μm (B) (TIF 2847 KB) [file 18_2022_4572_MOESM2_ESM.tif]

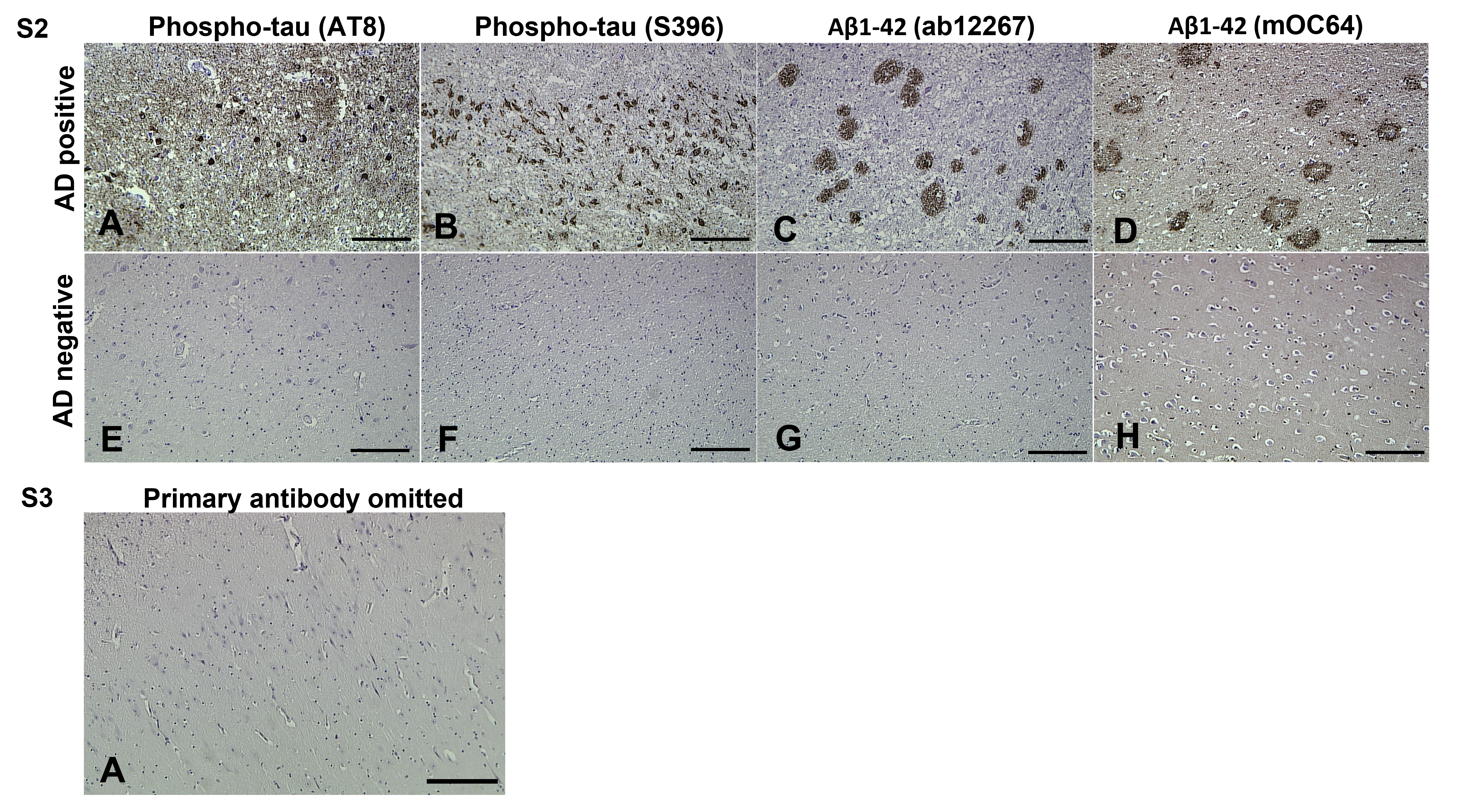

Supplement: Supplementary file 3 — Supplementary file3 Fig. S2 Immunohistochemistry of AD positive (top row) and AD negative (bottom row) human control tissue. The anti-phospho tau antibody, AT8 (A & E), the anti-phospho tau antibody, S396 (B & F), the anti-Aβ1–42, ab12267 (C & G), and the anti-Aβ1–42 mOC64 antibody (D & H). Scale bars represent 200 µm (A-H). Fig. S3 Immunohistochemical control section from the entorhinal cortex of a sheep > 5 years of age. The primary antibody (anti-Aβ1–42 mOC64, as seen in Fig. 3; A) has been omitted. Scale bar represents 200 µm (TIF 20936 KB) [file 18_2022_4572_MOESM3_ESM.tif]

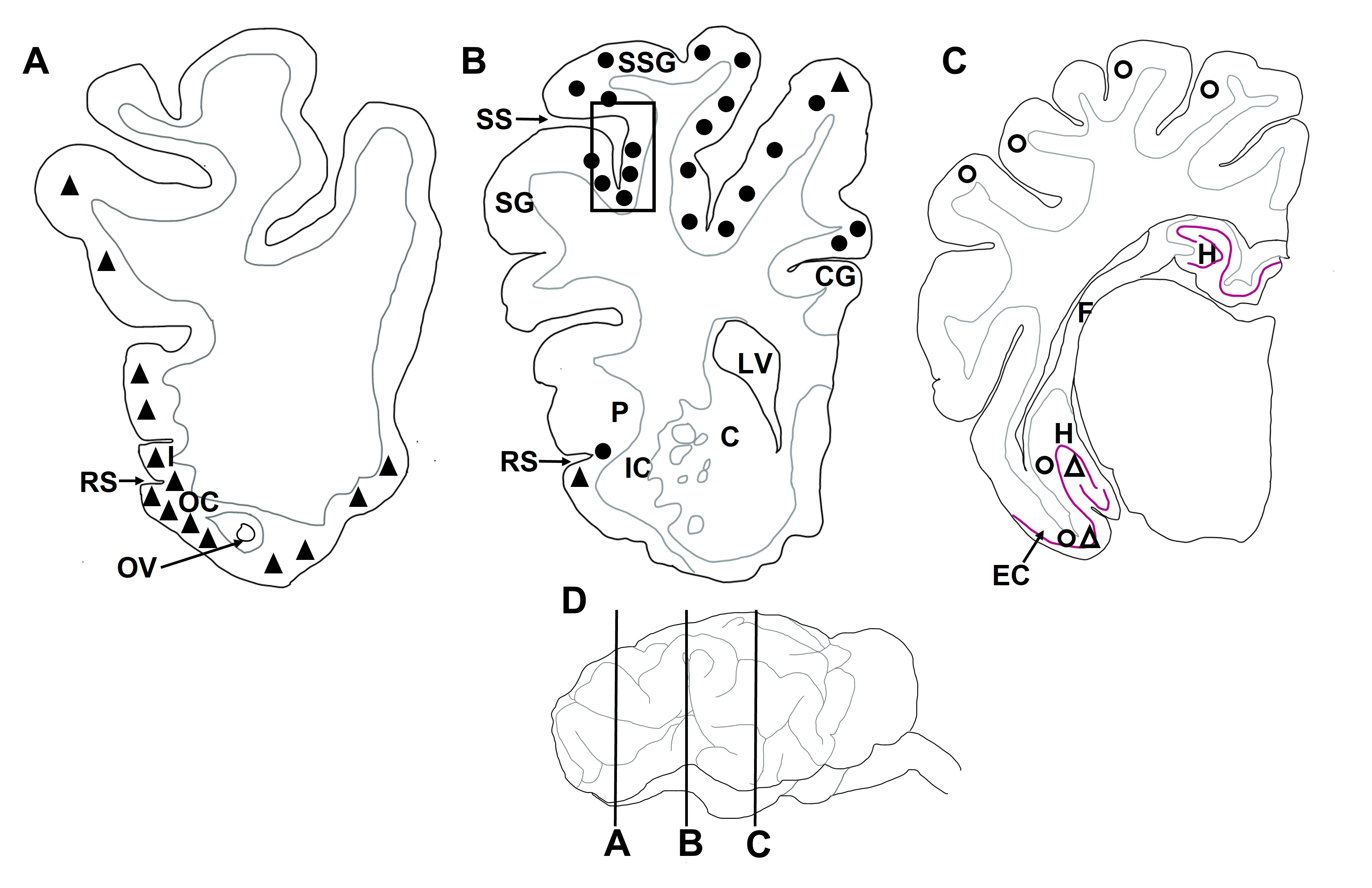

Supplement: Supplementary file 4 — Supplementary file4 Fig. S4: Schematic drawing of coronal sections of the sheep brain, representing the rostro-caudal distribution of AD-like pathologies in very old study pilot sheep (A; P1, B; P2), and stock sheep > 5 years of age (C). The left hemisphere viewed from the rostral aspect is illustrated. The approximate rostro-caudal positions of the coronal sections are illustrated in D. Solid triangles represent mature neurofibrillary tangles labelled with the anti-phospho-tau antibody AT8. Open triangles represent pre-neurofibrillary tangles and neuronal processes labelled with the anti-phospho-tau antibodies AT8 and S396. Solid circles represent diffuse plaques labelled with the anti-Aβ1–42 antibody ab12267. Open circles represent intra-neuronal labelling identified using the anti-Aβ1–42 antibody mOC64. The area enclosed by the box (B) represents the approximate location of Fig. 2; A. All gyri and sulci are labelled using the stereotaxis atlas of the ovine brain developed by Ella et al. [27]. C, caudate nucleus; CG, cingulate gyrus; EC, entorhinal cortex; F, fimbria; H, hippocampus; I, insular cortex; IC, internal capsule; LV, lateral ventricle; OC, olfactory cortex; OV, olfactory ventricle; P, putamen; RS, rhinal sulcus; SG, sylvian gyrus; SS, suprasylvian sulcus; SSG, suprasylvius gyrus (TIF 31288 KB) [file 18_2022_4572_MOESM4_ESM.tif]
