# Supplementary material for: Characterization of microtubule-associated protein tau isoforms and Alzheimer’s disease-like pathology in normal sheep (Ovis aries): relevance to their potential as a model of Alzheimer’s disease
Source: Cell Mol Life Sci. 2022 Oct 21;79(11):560. doi: 10.1007/s00018-022-04572-z (PMC9587068; doi:10.1007/s00018-022-04572-z)
Supplement: Supplementary file 5 — Supplementary file5 (PDF 164 KB) [file 18_2022_4572_MOESM5_ESM.pdf]

Characterization of microtubule associated protein tau isoforms and Alzheimer's disease-like pathology in normal sheep (*Ovis aries*) - relevance to their potential as a model of Alzheimer's disease.

Authors: Emma S. Davies[a], Russell M. Morphew[a], David Cutress[a], A. Jennifer Morton[b], Sebastian McBride[a]

[a] Department of Life Sciences, Aberystwyth University, Aberystwyth, Wales.

[b] Department of Physiology, Development and Neuroscience, University of Cambridge, Cambridge, UK

Correspondence: sdm@aber.ac.uk, +44 (0) 1970 621633, Institute of Biological, Environmental and Rural Sciences, Aberystwyth University, Aberystwyth, Wales.

Email: Emma S. Davies; esd5@aber.ac.uk, Russel M. Morphew; rom@aber.ac.uk, David Cutress; djc25@aber.ac.uk, A. Jennifer Morton; ajm41@cam.ac.uk, Sebastian McBride; sdm@aber.ac.uk

```
Human  MAEPRQEFEVMEDHA-GTYGLGDRKDQGGYTMHQDQEGDTDAGLKESPLQ     49
Mouse  MADPRQEFDTMEDHA-GDYTLL-----------QDQEGDMDHGLKESPPQ     38
Sheep  MAEPRQEFDVMEDHAQGDYTL------------QDHEGDMEPGLKESPLQ     38

Human  TPTEDGSEEPGSETSDAKSTPTAEDVTAPLVDEGAPGKQAAAQPHTEIPE     99
Mouse  PPADDGAEEPGSETSDAKSTPTAEDVTAPLVDERAPDKQAAAQPHTEIPE     88
Sheep  TPADDGSEEPGSETSDAKSTPTAEDATAPLVDEGALGEQAAAQAPTEIPE     88

Human  GTTAEEAGIGDTPSLEDEAAGHVTQARMVSKSKDGTGSDDKKAKGADGK-    148
Mouse  GITAEEAGIGDTPNQEDQAAGHVTQARVASK--DRTGNDEKKAKGADGKT    136
Sheep  GTTAEEAGIGDTSNLEDQAAGHVTQARMVSKGKDGTGPDDKKAKGADGKP    138

Human  -TKIATPRGAAPPGQKGQANATRIPAKTPPAPKTPPSSGEPPKSGDRSGY    197
Mouse  GAKIATPRGAASPAQKGTSNATRIPAKTTPSPKTPPGSGEPPKSGERSGY    186
Sheep  GTKIATPRGAAPPGQKGQANATRIPAKTTPTPKTSPGTGESGKSGDRSGY    188

Human  SSPGSPGTPGSRSRTPSLPTPPTREPKKVAVVRTPPKSPSSAKSRLQTAP    247
Mouse  SSPGSPGTPGSRSRTPSLPTPPTREPKKVAVVRTPPKSPSASKSRLQTAP    236
Sheep  SSPGSPGTPGSRSRTPSLPTPPTREPKKVAVVRTPPKSPSAAKSRLQAAP    238

Human  VPMPDLKNVKSKIGSTENLKHQPGGGKVQIINKKLDLSNVQSKCGSKDNI    297
Mouse  VPMPDLKNVRSKIGSTENLKHQPGGGKVQIINKKLDLSNVQSKCGSKDNI    286
Sheep  GPMPDLKNVKSKIGSTENLKHQPGGGKVQIINKKLDLSNVQSKCGSKDNI    288

Human  KHVPGGGSVQIVYKPVDLSKVTSKCGSLGNIHHKPGGGQVEVKSEKLDFK    347
Mouse  KHVPGGGSVQIVYKPVDLSKVTSKCGSLGNIHHKPGGGQVEVKSEKLDFK    336
Sheep  KHVPGGGSVQIVYKPVDLSKVTSKCGSLGNIHHKPGGGQVEVKSEKLDFK    338

Human  DRVQSKIGSLDNITHVPGGGNKKIETHKLTFRENAKAKTDHGAEIVYKSP    397
Mouse  DRVQSKIGSLDNITHVPGGGNKKIETHKLTFRENAKAKTDHGAEIVYKSP    386
Sheep  DRVQSKIGSLDNITHVPGGGNKKIETHKLTFRENAKAKTDHGAEIVYKSP    388

Human  VVSGDTSPRHLSNVSSTGSIDMVDSPQLATLADEVSASLAKQGL          441
Mouse  VVSGDTSPRHLSNVSSTGSIDMVDSPQLATLADEVSASLAKQGL          430
Sheep  VVSGDTSPRHLSNVSSTGSIDMVDSPQLATLADEVSASLAKQGL          432
```

Fig. S5 Multiple amino acid alignment of the longest human (NP_005901.2), murine (NP_001033698.1) and ovine (XP_027830182.1) tau protein sequences. Amino acids unique to the sheep are highlighted in bold red type. Major phosphorylation sites identified in humans are indicated by bold purple type. Conserved KXGS motifs which can be phosphorylated my multiple kinases are indicated by bold black type. Conserved PGGG sequences which facilitate the formation of type II β-turns and β-hairpin structures are indicated by bold blue type.
